# Genomics Armed With Diversity Leads the Way in *Brassica* Improvement in a Changing Global Environment

**DOI:** 10.3389/fgene.2021.600789

**Published:** 2021-02-18

**Authors:** Nur Shuhadah Mohd Saad, Anita A. Severn-Ellis, Aneeta Pradhan, David Edwards, Jacqueline Batley

**Affiliations:** School of Biological Sciences Western Australia and UWA Institute of Agriculture, University of Western Australia, Perth, WA, Australia

**Keywords:** *Brassica*, plant genomics, crop improvement, molecular breeding, food security

## Abstract

Meeting the needs of a growing world population in the face of imminent climate change is a challenge; breeding of vegetable and oilseed *Brassica* crops is part of the race in meeting these demands. Available genetic diversity constituting the foundation of breeding is essential in plant improvement. Elite varieties, land races, and crop wild species are important resources of useful variation and are available from existing genepools or genebanks. Conservation of diversity in genepools, genebanks, and even the wild is crucial in preventing the loss of variation for future breeding efforts. In addition, the identification of suitable parental lines and alleles is critical in ensuring the development of resilient *Brassica* crops. During the past two decades, an increasing number of high-quality nuclear and organellar *Brassica* genomes have been assembled. Whole-genome re-sequencing and the development of pan-genomes are overcoming the limitations of the single reference genome and provide the basis for further exploration. Genomic and complementary omic tools such as microarrays, transcriptomics, epigenetics, and reverse genetics facilitate the study of crop evolution, breeding histories, and the discovery of loci associated with highly sought-after agronomic traits. Furthermore, in genomic selection, predicted breeding values based on phenotype and genome-wide marker scores allow the preselection of promising genotypes, enhancing genetic gains and substantially quickening the breeding cycle. It is clear that genomics, armed with diversity, is set to lead the way in *Brassica* improvement; however, a multidisciplinary plant breeding approach that includes phenotype = genotype × environment × management interaction will ultimately ensure the selection of resilient *Brassica* varieties ready for climate change.

## Introduction

Predictions of exponential increases in the world population and climate change are forcing re-evaluation of efforts in addressing the demand for global food security. Plant crops account for more than 80% of the diet consumed by the human population, and the production of edible crops has thereby dominated almost half of the world's available land mass since the beginning of the twenty-first century (Leff et al., 2004; Herrera and Garcia-Bertrand, 2018). The Industrial and Green Revolutions have furthermore shaped the ways that these commodities are managed for efficiency and commercialization. The next revolution will require crop improvement not just to curb world hunger but also to address sustainability in the face of biotic and abiotic stresses triggered by the impending climate change. It is estimated that staple crop yield must improve by 70–110% to feed the predicted 10 billion population by 2050 (Saini et al., 2020). Crop projections and modeling studies have suggested that climate change may have already been responsible for a small yearly decrease in yield and calories in certain geographic regions (Ray et al., 2019). More recently, the coronavirus disease 2019 (COVID-19) pandemic has emphasized food security in terms of short-term and local supply (Cappelli and Cini, 2020). While food shortages are not a wide set concern as yet, a prolonged crisis could interfere with the current complex food supply network (de Paulo Farias and dos Santos Gomes, 2020; Siche, 2020). Globalized food distribution, though highly profitable, highlighted the critical gap in local production, closer to consumers and less likely affected by international restrictions (Cappelli and Cini, 2020). Meeting local population demand, while remaining sustainable in a shorter food supply chain, may yet have introduced another facet to food security in the post-COVID-19 era. Predicted environmental variation including rising temperatures and increases in carbon dioxide emissions could result in a drier atmosphere and an increase in evapotranspiration (Ficklin and Novick, 2017). In addition to water, most biological processes are temperature sensitive, and climate change is therefore undoubtedly going to affect all crop performance (Dusenge et al., 2019). As a result, resilience to abiotic stresses, such as heat, drought, and salinity, will become traits that are highly desirable in future crop improvement strategies. Reactive nitrogen plays an important role in plant growth, crop yield, and subsequently human nutrition (Dreccer et al., 2000). Alongside their benefits, agricultural practices such as nitrogen fertilizer application and nitrogen fixing crops have the potential to disturb the global nitrogen cycle and adversely affect human health (Townsend et al., 2003; Bodirsky et al., 2012). These practices, if poorly managed, can contribute significantly toward the release of nitrous oxide into atmosphere, which negatively affects the protective ozone layer and advances climate change (Crutzen and Ehhalt, 1977). In an effort to solve the global nitrogen challenge, Houlton et al. (2019) propose, amongst other things, the improvement of nitrogen-use efficiency in crop production. This could be achieved by altering fertilizers and fertilizer application practices, boosting soil health to promote nitrogen uptake, and developing improved crop varieties that efficiently utilize nitrogen (Houlton et al., 2019).

The genus *Brassica* consists of extensively agronomically diverse species. Oilseed canola include *Brassica rapa, Brassica napus*, and *Brassica juncea* varieties with internationally defined erucic acid and glucosinolate contents (Sharafi et al., 2015). Vegetable Brassicas include *B. rapa* ssp. *rapa* (turnip), ssp. *oleifera* (turnip rape), ssp. *chinensis* (pak choi/bok choy), and ssp. *pekinensis* (Chinese cabbage); *Brassica oleracea* ssp. *capitata* (cabbages), var. *italica* (broccoli), var. *botrytis* (cauliflower), ssp. *gemmifera* (Brussels sprouts), and ssp. *alboglara* (Chinese kale); and *B. napus* var. *napobrassica* (swede/rutabaga) (Cheng et al., 2016). Mustard *Brassica* include *Brassica nigra* (black mustard), *Brassica carinata* (Ethiopian mustard), and *B. juncea* (Indian mustard). The U triangle summarized the interspecific hybridization events between diploid progenitors *B. rapa* (AA), *B. nigra* (BB), and *B. oleracea* (CC) resulting in polyploidy, *B. juncea* (AABB), *B. napus* (AACC), and *B. carinata* (BBCC) (Nagaharu et al., 1935; Snowdon et al., 2002).

Brassicas are cultivated on a worldwide scale, and it is therefore almost a certainty that forecasted environmental changes would affect the crop (Francisco et al., 2017). Combined with the abiotic stresses, biotic stress is another challenge facing global *Brassica* production. Bebber et al. (2013) combined observation data and mathematical equation to project pest distribution and proposed that pathogen and pests affecting global crops are moving polewards as the temperature rises. Pathogens of *Brassica* oilseed and vegetable crops, such as *Leptosphaeria maculans* (blackleg or stem canker), *Alternaria brassicae* (Alternaria blight), *Albugo candida* (white rust), *Pseudocercosporella capsellae* (white leaf spot), *Plasmodiophora brassicae* (club root), and *Sclerotinia sclerotiorum* (Sclerotinia stem rot), extensively affect yield, seed quality, and crop development (Murray and Brennan, 2012). Breeding *Brassica* varieties that can withstand the pressures of a changing environment is perhaps the best strategy in ensuring sustainability.

Decreasing nutrient content of modern fruit and vegetable cultivars has raised concern in recent studies conducted in the USA and UK, predicting the future need for agricultural bio-fortification (Mayer, 1997; Davis et al., 2005; White and Broadley, 2005; Davis, 2009). Davis coined the term “genetic dilution effect” in 2005 after observing broccoli hybrids (*B. oleracea* var. *italica*) accumulating denser heads without the obvious proportional increase in nutrients (Davis et al., 2005). This is a result of breeding and selection of varieties based on yield and productivity, while overseeing the importance of nutrient content. The World Health Organization considers micronutrient deficiency as a major health challenge especially in developing and poor countries (Khush et al., 2012). Bio-fortification is a cost-effective and sustainable strategy in addressing malnutrition; however, this requires suitable genetic diversity within the genepool to be valuable in breeding (Garg et al., 2018; Kumar et al., 2020).

Available genetic diversity still constitutes the foundation of all breeding efforts. Elite varieties, land races, and crop wild species are important resources of useful variation that can be introgressed, re-introduced, or manipulated to obtain the required biotic and abiotic resilience in *Brassica* crops (Dwivedi et al., 2017). The identification and exploitation of suitable variation are crucial for crop improvement (Hu et al., 2018) and can be elucidated at the genome scale (Varshney et al., 2018). Genomics can, in addition, contribute toward unraveling the genetic origin and molecular pathways involved in biotic and abiotic stress tolerance traits. Complete and accurate understanding of the ancestry of the *Brassica* species will assist in the tracking and exploitation of genetic inheritance of useful traits (Bancroft et al., 2011).

Crop improvement has always been a co-evolutionary process between humans and edible plants (Harlan, 1992); changes in plants brought about by cultivation allowed changes in human populations to take place. Plant breeding has largely relied on conventional breeding methods based on phenotypic selection. However, it is in doubt whether conventional breeding approaches alone would be adequate in addressing the impending challenges. During the course of the last three decades, genomics has become an integral part of all life sciences. Rapid advances in sequencing tools followed by cost reductions, as well as the development of high-throughput genotyping techniques, have led to advances in trait mapping, functional characterization, and ultimately crop improvement through genomic selection (GS) (Nepolean et al., 2018).

Without available genetic diversity, the introduction of genes may present a suitable solution. Weed and insect control using genetically modified (GM) crops has assisted farmers worldwide in attaining higher yields with fewer resources (Zhang et al., 2016). Cauliflower (Lu et al., 2006), mustard (Hong et al., 2002), and canola (Garg et al., 2018) have been subjected to transgenic bio-fortification efforts for beta-carotene, gamma-linoleic acid, and phytate degradation, respectively. Despite these efforts, so far, only Phytaseed canola has been released by BASF in the USA. Health concerns, consumer skepticism, and long and expensive regulatory processes are restricting the release of transgenic crops (Watanabe et al., 2005). Recent developments in genome editing (GE) provide an option to alter or introduce specific genes in order to obtain the desired trait expression. Oligo-directed mutagenesis (ODM), programmable sequence-specific nucleases (SSNs), and base-editing tools allow the precise creation of insertions/deletions (indels) or even the introduction of a complete sequence at a predetermined target location within the genome (Scheben et al., 2017).

To further address the pressing demands on crop improvement, an accelerated rate of genetic gain is required. The implementation of GS can fast-track the progress in crop breeding (Wang X. et al., 2018). In GS, predicted breeding values based on phenotype and genome-wide marker scores allow the preselection of promising genotypes, thereby substantially quickening the breeding cycle and enhancing genetic gains (Heffner et al., 2009). Further optimization of mating strategies is essential to prevent inbreeding and ensure long-term genetic gain (Allier et al., 2019).

**Figure 1:**
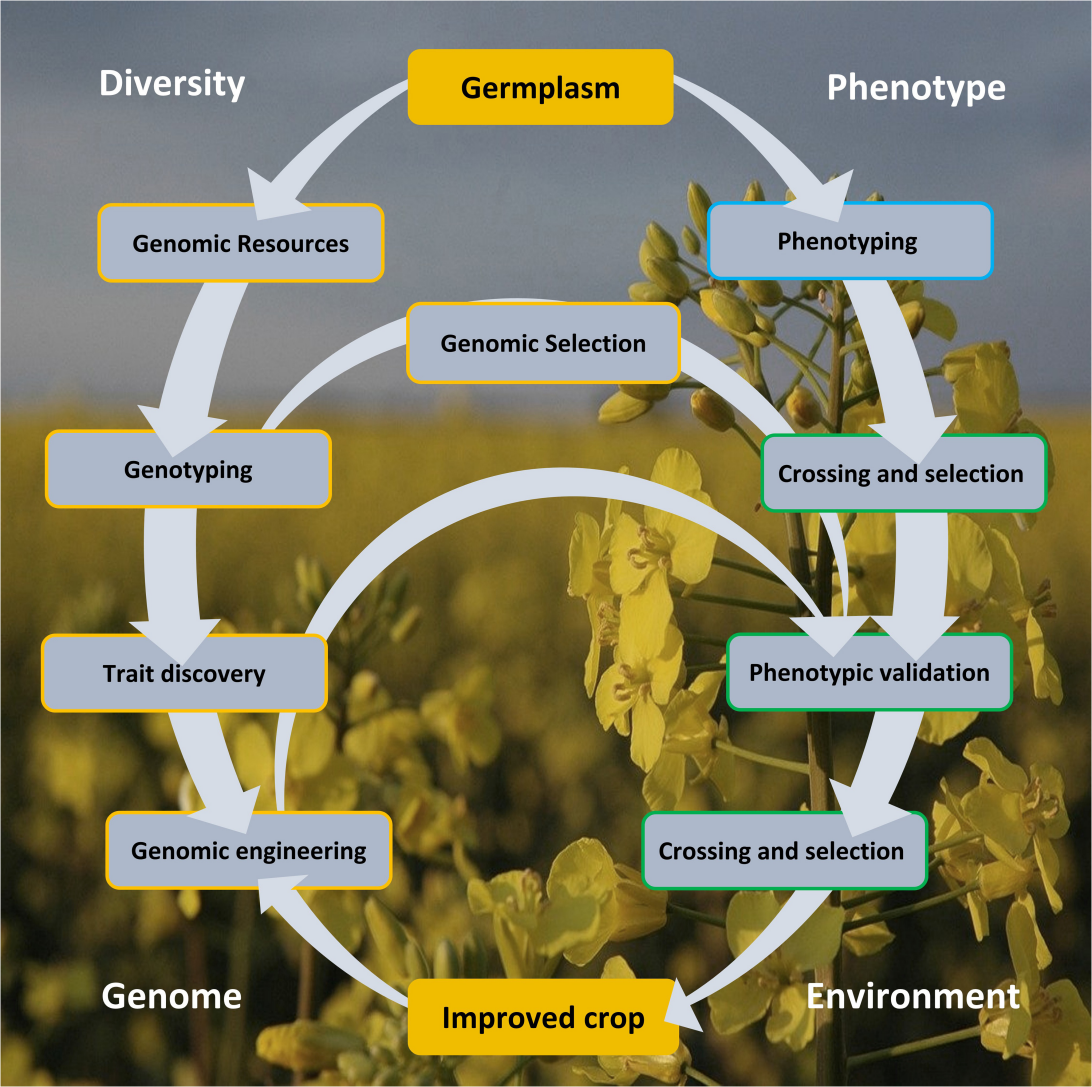
The crop improvement cycle involves the exploitation of genomic diversity and phenotypic variability. These approaches offered a rapid and cost-effective lab-to-field and back loop, centered on arming the genomic arsenal by capitalizing on the variation in the germplasm resource, as defined in the genotype and from trait association. Genomic selection and engineering accelerated crop improvement, circumventing a more laborious and time-consuming conventional crossing and selection approach. Genomic engineering strategies also offer a more precise manipulation for improved crop development. The improved crop must be climate resilient and adaptive to the extreme environmental changes.

Genomics armed with diversity is currently leading the way in crop improvement. Here, we review aspects of the *Brassica* crop improvement cycle (Figure 1) illustrating the importance of genetic diversity, creation of genomic resources, its exploitation in aid of trait discovery, and GE and GS of *Brassica*.

## Estimation and Conservation of Diversity in Germplasm

Crop breeding is required to be sustainable and to adapt promptly in the face of abiotic and biotic environmental changes (Zhang and Batley, 2020). Most modern crops were developed through repeated cycles of selection, “filtering out” varieties with desirable agronomic traits from ancestral wild species. However, potentially valuable genetic variation is often lost in the process, resulting in lowered trait heritability and increased genetic homogeneity (Rahman, 2013). Comparative population genomics can be used to identify these selective sweeps, or bottlenecks, and potential loci under selection (Slatkin, 2008). An example is the allotetraploid *Brassica napus* (AACC, 2n = 38), which originated from spontaneous interspecific hybridization events between *Brassica rapa* (AA, 2n = 20) and *Brassica oleracea* (CC, 2n = 18) (Chalhoub et al., 2014). Cultivated *B. napus* have been under intense selection over the past decades, which has led to a severe genetic bottleneck in the species (Becker et al., 1995). This is reflected by the extent of highly conserved regions found between the genomes of *B. napus* accessions found in almost all major genepools (Werner et al., 2018). Accordingly, the decline of allelic variation and genetic diversity was reported in Canadian (Fu and Gugel, 2010) and Australian spring canola breeding initiatives (Cowling, 2007).

The key to sustainable crop improvement in the face of climate change, and increased pressure from pests and diseases, is maintaining this diversity. Diversity within germplasm provides breeders with valuable material to enable the selection of parental lines, exploitation of heterosis, and the expansion of breeding pools (Yousef et al., 2018). Heritable variation present within the crop germplasm is therefore essential for efficient breeding programs. Characterizing the genetic diversity allows breeders to select alleles at loci of interest and identify trait-associated markers suitable for introgression into new varieties. The assessment of germplasm diversity furthermore assists in the optimization of conservation strategies of germplasm collections (Rao and Hodgkin, 2002). This was highlighted in the study conducted by Yousef et al. (2018), in which cauliflower (*B. oleracea* var. *botrytis*) accessions originating from 26 countries were grouped into two major groups representing the two genebanks from which the accessions were obtained and not the country of origin. In this case, composition and accession type influenced the level of diversity and contributed toward the differentiation between the genebanks. Routine monitoring of genetic diversity in *ex situ* germplasm collections might therefore be essential to prevent potential loss of genetic diversity.

Wild species related to agricultural crops [crop wild relatives or (CWRs)] and landraces offer an attractive alternative source of variation (Dempewolf et al., 2017; Khan et al., 2020). Many of these unique resources are currently available from genebanks and seed repositories (Tanksley and McCouch, 1997). Introgression from CWRs or landraces can broaden the genetic base for modern breeding programs and can contribute to sought-after characteristics associated with relative tolerance to extreme environments and disease resistance. Despite the vast genetic potential locked up in these resources, their utilization is hampered by inconsistent documentation, unintentional duplications, and a lack of available genetic information (Singh N. et al., 2019; Zhang and Batley, 2020). Combining available phenotypic and geographical descriptors with genomic sequence information, perhaps in the form of a universal molecular passport, could facilitate the selection of useful genetic variation and its use in breeding programs (Mascher et al., 2019; Singh N. et al., 2019).

Plant genetic resources, including CWRs in the wild, are considered under threat and in need of conservation (Dempewolf et al., 2017). Climate change, along with habitat fragmentation due to human activity, is predicted to result in drastic plant population declines and even result in extinction (Jump and Peñuelas, 2005). The creation of protected areas, efforts to reduce pollution and legal frameworks to protect endangered species, such as the Convention on International Trade in Endangered Species of Wild Fauna and Flora (CITES), may aid in the prevention of the decline or loss of biodiversity. Genetic approaches over time have contributed toward the estimation of biodiversity and prioritization of conservation efforts. However, genome-scale data and associated high-density markers can improve estimations of genetic diversity and population structure. Transitioning from a genetics to conservation genomics approach is expected to have a positive impact on future conservation recommendations and policies (Supple and Shapiro, 2018).

## Creation of Genomic Resources in *Brassica* Species

The smaller Brassicaceae genome from *Arabidopsis thaliana* was the first to be sequenced and benefited progress in *Brassica* sequencing given the high degree of genomic conservation between *A. thaliana* and *Brassica* species. In addition, DNA sequencing technology becomes more affordable with longer reads and higher throughput available at a fraction of the cost (Marri et al., 2018). These developments provided the opportunity to improve *de novo* genome assembly, increase mapping certainty, and identify structural variants (SVs) (Amarasinghe et al., 2020). Furthermore, re-sequencing efforts have increased over time to resolve challenges and gaps in current studies, especially in complex and agronomically significant crops like *Brassica napus*.

As technology progresses, more advanced genomic tools become available, expanding the range of analysis and exploitation possibilities of germplasm resources. The PlabiPD database (https://www.plabipd.de/) maintains an updated list of sequenced plant species, including Brassicas, which can be visualized phylogenetically or temporally. Available genomic resources have played an important role in the advancement of breeding programs throughout the world in cereals, legumes, oilseeds, and even ornamental crops. Hence, here we examine the development and availability of nuclear and organellar genome resources generated in Brassicas.

### Nuclear Genome

Draft genomes and pangenome assemblies have been created for five domesticated and a wild *Brassica* species (Table 1). More broadly, 27 members of the Brassicaceae family have been sequenced, including three *Arabidopsis* (The Arabidopsis Genome Initiative, 2000; Hu T. T. et al., 2011; Akama et al., 2014; Briskine et al., 2017; Michael et al., 2018), three *Capsella* (Slotte et al., 2013; Kasianov et al., 2017), three *Eutrema* (Yang et al., 2013; Guo et al., 2018), and two *Raphanus* species (Kitashiba et al., 2014; Moghe et al., 2014; Shirasawa et al., 2020). The earlier *Brassica* genome assemblies focused on elite cultivars and combined Illumina paired-end reads with bacterial artificial chromosome (BAC)-end sequences to construct scaffolds and build high-quality assemblies. Markers from genetic maps were used, in addition, to merge assemblies and anchor scaffolds to pseudo-chromosomes. The genomes were either assembled *de novo* or based on a reference genome of the closest relative. The assembly of *B. napus* followed an earlier approach for allopolyploids by sequencing the diploid progenitor genome, similar to the methods used in the assembly of the strawberry and cotton genomes (Tennessen et al., 2014; Ming and Man Wai, 2015; Zhang et al., 2015).

**Table 1:** List of domesticated and wild *Brassica* species nuclear genome assemblies.

**Species**	**Current status**	**Assembly size (bp)**	**Number of protein-coding genes**	**Genomic tools**	**References**
*Brassica rapa* (2n = 10)	Chinese cabbage ssp. *pekinensis* cv. Chiifu 401–42, *de novo* assembly (version 1.5)	283.80 Mb	41,174	Illumina GAII short, medium, long insert libraries, BAC-end Sanger sequences, EST data, and genetic maps	Wang et al., 2011
	Japanese turnip ssp. *rapa* cv. DH-VT117, reads mapped to *B. rapa* Wang et al. (2011) reference genome	282.93 Mb	40,708	Illumina Hiseq2000 short, medium, long insert libraries	Lin et al., 2014
	Rapid cycling oil-like inbred line cv. RC-144, reads mapped to *B. rapa* Wang et al. (2011) reference genome	282.69 Mb	40,506		
	Chinese cabbage ssp. *pekinensis* cv. Chiifu 401–42, *de novo* assembly (version 2.0/2.5)	389.20 Mb	48,826	Illumina and PacBio sequencing, and genetic maps	Cai et al., 2017
	Chinese cabbage ssp. *pekinensis* cv. Chiifu 401–42, *de novo* assembly (version 3.0)	353.14 Mb	45,985	Illumina HiSeq4000 short reads, PacBio SMRT long reads, BioNano optical mapping, Hi-C sequencing, and genetic map	Zhang L. et al., 2018
	Yellow sarson ssp. *trilocularis* cv. Z1, *de novo* assembly	401.92 Mb	46,721	Illumina HiSeq2500 short, medium, long insert libraries, Oxford Nanopore long reads, BioNano optical mapping, and genetic map	Belser et al., 2018
	Chinese cabbage ssp. *chinensis* cv. NHCC001 (Suzhouqing), *de novo* asssembly	405.33 Mb	48,158	Illumina short reads, PacBio Sequel SMRT long reads, and Hi-C sequencing	Li et al., 2020
*Brassica oleracea* (2n = 18)	Chinese kale ssp. *capitata* cv. TO1000DH3, *de novo* assembly	488.60 Mb	59,225	Illumina HiSeq2000, Roche 454 GS-FLX Titanium sequencing, and BAC-end sequences, and genetic map	Parkin et al., 2014
	Chinese kale ssp. *capitata* cv. 02-12, *de novo* assembly	539.90 Mb	45,758	Illumina GAII shotgun sequencing combined with Roche 454 GS-FLX Titanium sequencing, BAC-end sequences, and genetic map	Liu et al., 2014
	Pangenome of nine varieties including cabbages, kale, Brussels sprout, Kohlrabi, cauliflowers, broccoli, and wild *Brassica macrocarpa*, with draft genome by Parkin et al. (2014) as reference	587.00 Mb	61,379	Illumina HiSeq2000 and HiSeq2500 short reads	Golicz et al., 2016
	Broccoli ssp. *botrytis italica* cv. HDEM, *de novo* assembly	554.97 Mb	61,279	Illumina HiSeq2500 short, medium, long insert libraries, Oxford Nanopore long reads, BioNano optical mapping, and genetic map	Belser et al., 2018
	Cauliflower var. *botrytis* cv. C-8	584.60 Mb	47,772	Illumina HiSeq and X Ten short reads, and PacBio Sequel SMRT long reads	Sun et al., 2019
	Cabbage var. *capitata* cv. D134 DH (commercially known as Zhonggan 18)	529.92 Mb	44,701	Illumina HiSeq2500 short reads, PacBio Sequel SMRT long reads sequencing, Illumina HiSeq X Ten and GemCode 10x Genomics short reads, and Hi-C sequencing	Lv et al., 2020
*Brassica napus* (2n = 38)	European winter-type cv. Darmor (version 4.1)	712.30 Mb	101,040	Roche 454-GS-FLX+ Titanium long reads, BAC-end sequences, and Illumina HiSeq short reads	Chalhoub et al., 2014
	European winter-type cv. Darmor (version 8.1)	616.70 Mb	80,382	Illumina HiSeq short, medium, and long insert libraries, and genetic map	Bayer et al., 2017
	European spring-type cv. Tapidor, *de novo* assembly	625.90 Mb	70,162		
	Asian semi-winter cv. ZS11, *de novo* assembly	854.98 Mb	90,731	Illumina HiSeq2500 short, medium, long insert libraries, BAC sequencing, and genetic maps	Sun et al., 2017
	Pangenome of 20 synthetic and 33 non-synthetic accessions based on Darmor v8.1 assembly	1,044 Mb	94,013	Illumina sequencing	Hurgobin et al., 2018
	Asian semi-winter cv. Ningyou 7, *de novo* assembly	994.00 Mb	104,179	Illumina, PacBio RS II, and Hi-C sequencing, and genetic map	Zou et al., 2019
	Pangenome of eight varieties (Westar, No2127, Zheyou7, Gangan, Shengli, Tapidor, Quinta, ZS11) and *de novo* assemblies of each genome	1,001–1,033 Mb	94,586–100,919	Illumina short reads, PacBio SMRT long reads, Hi-C sequencing, and BioNano optical mapping	Song et al., 2020
	European winter-type cv. Express 617	925.00 Mb	89,857	Illumina short reads, PacBio SMRT and Oxford Nanopore long reads, BioNano optical mapping, and genetic map	Lee et al., 2020
	European winter-type cv. Darmor (version 10)	924.00 Mb	108,190	Illumina HiSeq2500 short reads, PromethION Oxford Nanopore long reads, optical mapping, and genetic map	Rousseau-Gueutin et al., 2020
	Asian semi-winter cv. ZS11	1,160 Mb	106,059	Illumina short reads, PacBio SMRT long reads, Hi-C sequencing, and genetic map	Chen et al., 2020
*Brassica juncea* (2n = 36)	var. *tumida* cv. T84-66 inbred line, *de novo* assembly	949.60 Mb	80,050	Illumina HiSeq short reads, PacBio SMRT long reads, BioNano optical mapping, and genetic map	Yang et al., 2016a
	cv. Varuna	922.00 Mb	101,959	Illumina HiSeq1000 short reads, PacBio RS II SMRT long reads, BioNano optical mapping, and genetic map	Paritosh et al., 2020
*Brassica nigra* (2n = 16)	cv. YZ12151 DH, *de novo* assembly	396.90 Mb	49,826	Illumina HiSeq short reads, BAC-end sequences, and genetic map	Yang et al., 2016a
	cv. Sangam (line BnSDH-1)	515.40 Mb	46,227	Illumina HiSeq1000 and MiSeq short reads, Oxford Nanopore long-read sequencing and optical mapping, and genetic map	Paritosh et al., 2020
	cv. Ni100 (short and long reads)	447.00 Mb (SR), 506.00 Mb (LR)	56,331 (SR), 59,877 (LR)	Illumina and Roche 454 short reads, GridION Oxford Nanopore long reads, and genetic map	Perumal et al., 2020
	cv. CN115125 (C2) (long reads)	537.00 Mb	67,030	Illumina HiSeq2500 short reads, MinION Oxford Nanopore long reads, Hi-C sequencing, and genetic map	
*Brassica cretica* (2n = 18)	Four individuals from isolated Grecian population of subsp. *cretica* and subsp. *nivea* assembled based on related *B. oleracea* Liu et al. (2014) reference	40.02–412.52 Mb	30,360	Illumina HiSeq2500 short reads	Kioukis et al., 2020

Brozynska et al. (2016) explored the progress of CWR sequencing efforts and noted fewer efforts compared with domesticated relatives, a trend also observed in *Brassica*. Higher levels of heterozygosity in CWRs, which can result in greater assembly difficulties, might be contributing toward the trend (Brozynska et al., 2016). Recently, the genomes of two wild diploid perennial *Brassica* C-genome species were sequenced (Golicz et al., 2016; Kioukis et al., 2020). *Brassica macrocarpa* and *Brassica cretica*, both native to Greece, are potential wild progenitors of *Brassica oleracea*, which was thereby used as reference in the assembly of the two CWRs (Branca et al., 2012).

Next-generation sequencing (NGS) technologies, although extensively and successfully used in genome assembly, are limited by their relatively short read lengths. Shortcomings include misassembly and gaps in long repeat regions, difficulties in detecting larger SVs, transcript isoforms, and haplotype phasing (Van Dijk et al., 2018). Long-read third-generation sequencing (TGS) technologies from Pacific Biosciences (PacBio) and Oxford Nanopore Technologies (ONT) allow less bias and more homologous coverage of the genome, thereby overcoming the challenges such as polyploidy and frequent repetitive elements. TGS and NGS are often combined with long-range mapping technologies (BioNano Genomics) and chromosome conformation capture (Hi-C) (Van Berkum et al., 2010), to enable the assembly of highly contiguous chromosome-level crop genome assemblies (Hu et al., 2018; Schreiber et al., 2018). Song et al. (2020) created eight high-quality *B. napus* reference genomes by integrating different combinations of Illumina, PacBio, Hi-C, and BioNano data. These high-quality *B. napus* reference genomes allowed the identification of SVs, including copy number variants (CNVs) and presence and absence variations (PAVs), and improve our understanding of the genome structure and genetic basis behind phenotype differentiation in *B. napus*.

Genomic comparison between two *B. napus* reference genomes by Bayer et al. (2017) highlighted the limitations of a single reference assembly, given the wealth of variation between individuals, concluding that the genetic diversity at a species level cannot be sufficiently captured by a single reference genome (Hurgobin and Edwards, 2017). The pangenome was “born” in an effort to capture the totality of diversity in a species or something broader (Vernikos, 2020). *B. oleracea* (Golicz et al., 2016) and *B. napus* (Hurgobin et al., 2018) were the first *Brassica* pangenomes to be published and investigated the diversity within the species. The second *B. napus* pangenome (Song et al., 2020) combined TGS technologies, including PacBio single-molecule real-time (SMRT) and ultralong nanopore sequencing. These pangenome assemblies demonstrated the prospect of uncovering and mining diversity within secondary crop genepools for crop improvement (Voss-Fels and Snowdon, 2016).

Affordability of sequencing is promoting a combination of *de novo* assembly and whole-genome re-sequencing (WGRS) efforts of wider genepools, including close relatives and CWRs in an effort to identify and explore useful genetic variation (Brozynska et al., 2016). While the pangenome represents the genomic makeup of a species (Tettelin et al., 2005), the super-pangenome includes the CWR genetic variability that has been lost due to domestication and breeding selection bottlenecks (Khan et al., 2020). This was well-demonstrated by the discovery of an abundance of unique genes upon the inclusion of the wild *Brassica macrocarpa*, in comparison with the other eight domesticated varieties, in the *B. oleracea* pangenome (Golicz et al., 2016). These genes form part of the pangenome's dispensable genome, and the findings yet again emphasize the value of the genetic variability captured within the wild *Brassica* spp. It is predicted that efforts to catalog and include more lines into pangenomes and super-pangenomes would probably never cease, thereby providing a constant contribution of valuable resources for crop improvement efforts in an ever-changing environment. Future exploration in genomic resources in *Brassica* will likely involve additional WGRS efforts and further pangenome studies to explore breeding histories and identify loci associated with important agronomic traits such as oil content and composition, seed quality, and disease resistance (Wang Y. et al., 2016; Lu et al., 2019; Dolatabadian et al., 2020; Gabur et al., 2020; Yan et al., 2020; Zhang et al., 2020).

### Organelle Genomes

In *Brassica*, the mitochondrial (mt) genome assembly predates that of the whole genome due to its significantly smaller size (~20 kbp) (Palmer and Herbon, 1988; Handa, 2003; Kode et al., 2005) and high copy number per cell (Lima et al., 2016) (Table 2). Due to its small size in comparison with other higher plants, the *Brassica* mt genome was used as an early model in the understanding the plant mt, structure, function, and content (Grewe et al., 2014). Comparative analysis of the mt genome can be used to study interspecific phylogenetic relationships (Darracq et al., 2011) and uncover the plant's evolutionary history (Xue et al., 2020). For example, the mt genomes of *Brassica rapa* subspecies with distinct morphologies were found to be highly conserved (Hatono et al., 2017). Furthermore, the rate of mutation for *Brassica* mt DNA was four times slower than that of the chloroplast (Cp) DNA (Palmer and Herbon, 1988). Xue et al. (2020) compared mt DNA of the six members of the U triangle (Nagaharu et al., 1935) and revealed that *B. oleracea* was undergoing the most mt genomic change, while the B-genomes containing *Brassica carinata* and *Brassica nigra* were identified as maternally more distantly related to the remaining *Brassica* accessions of the U triangle (Xue et al., 2020). It was also suggested that *Sinapis arvensis* could have been misclassified based on both phylogenetic and mt genomic organization, placing it within the *Brassica* species and sister to the *B. nigra–B. carinata* lineage (Sang et al., 2020; Xue et al., 2020). Sang et al. (2020) also confirmed that the genome structure and evolutionary analysis of the *S. arvensis* organellar genomes were more similar to those of *B. nigra* and *B. carinata*. In addition, mt genes, encoding putative proteins with transmembrane domains, were discovered, which may explain the alloplasmic male sterility of novel cytoplasmic male sterility (CMS) derived from somatic cell hybridization between *B. napus* and *S. arvensis*.

**Table 2:** List of domesticated *Brassica* species organelle genome sequences.

**Genome type/species**	**Data accession**	**Size (bp)**	**Number of protein-coding gene**	**Genomic tools**	**References**
**Mitochondrial genome**					
*Brassica rapa* (Chinese cabbage, turnip)	N/A	~218 kb	Mapped 12 genes (61 kb of genome was highly transcribed)	Physical map using *Pst*I, *Sal*I, *Bgl*I, *Kpn*I, and *Nru*I restriction endonucleases and cloning	Palmer and Shields, 1984; Makaroff and Palmer, 1987
*B. rapa* cv. Purple Top White Globe (turnip)	N/A	~218 kb	N/A	Physical map using *Sal*I and *Pst*I restriction endonucleases and cloning, and comparative mapping with 15 other enzymes	Palmer and Herbon, 1988
*B. rapa* cv. Suzhouqing	JF920285	219,747	34 (3 rRNA, 18 tRNA)	Roche 454 GS-FLX DNA pyrosequencing, PCR, and Sanger sequencing	Chang et al., 2011
*B. rapa* var. Oushou hakusai (Chinese cabbage)	AP017996	219,775	34 (3 rRNA, 18 tRNA)	Roche 454 GS-FLX DNA pyrosequencing, PCR, and Sanger sequencing	Hatono et al., 2017
*B. rapa* var. Chusei shiroguki sensuji kyomizuna (mizuna)	AP017997			Illumina MiSeq, PCR, and Sanger sequencing	
*Brassica napus* cv. American Purple Top (rutabaga)	N/A	~221 kb	N/A	Physical map using *Sal*I and *Pst*I restriction endonucleases and cloning, and comparative mapping with 15 other enzymes	Palmer and Herbon, 1988
*B. napus* cv. Westar	AP006444	221,853	34 (3 rRNA, 17 tRNA)	Long range PCR and sequencing	Handa, 2003
*B. napus* CMS plant (Polima)	FR715249	223,412	34 (3 rRNA, 18 tRNA)	Cycle sequencing, PCR, and Sanger sequencing	Chen et al., 2011
*Brassica oleracea* (cauliflower)	N/A	~217 kb	N/A	Physical map using *Sal*I, *Kpn*l, and *Bgl*l restriction endonucleases and cloning	Chétrit et al., 1984
*B. oleracea* cv. Brunswick (cabbage)	N/A	~219 kb	N/A	Physical map using *Sal*I and *Pst*I restriction endonucleases and cloning, and comparative mapping with 15 other enzymes	Palmer and Herbon, 1988
*B. oleracea* cv. 08C717	JF920286	360,271	56 (4 rRNA, 35 tRNA)	Roche 454 GS-FLX DNA pyrosequencing, PCR, and Sanger sequencing	Chang et al., 2011
*B. oleracea* var. *botrytis* (cauliflower)	N/A	219,962	33 (3 rRNA, 18 tRNA)	Illumina GAIIx, PCR, and Sanger sequencing	Grewe et al., 2014
*B. oleracea* var. Fujiwase (cabbage)	AP012988	219,952	34 (3 rRNA, 17tRNA)	Roche 454 GS-FLX DNA pyrosequencing, PCR, and Sanger sequencing	Tanaka et al., 2014
*B. oleracea* var. *capitata* cv. 4119 (cabbage)	KU831325	219,975	34 (3 rRNA, 19 tRNA)	Illumina HiSeq2000, PCR, and Sanger sequencing	Yang K. et al., 2018
*Brassica nigra* (black mustard)	N/A	~231 kb	N/A	Physical mapping using BstEII–*Bgl*I and *Pst*I restriction endonucleases and cloning	Palmer and Herbon, 1986
*B. nigra* cv. Ni-138	AP012989	232,145	33 (3 rRNA, 17 tRNA)	Roche 454 GS-FLX DNA pyrosequencing, PCR, and Sanger sequencing	Yamagishi et al., 2014
*B. nigra* (black mustard)	KP030753	232,407	33 (3 rRNA, 19 tRNA)	Illumina HiSeq2500, PCR, and Sanger sequencing	Yang et al., 2016b
*Brassica juncea* cv. Jiangpu-yejiecai	JF920288	219,766	34 (3 rRNA, 18 tRNA)	Roche 454 GS-FLX DNA pyrosequencing, PCR, and Sanger sequencing	Chang et al., 2011
*Brassica carinata* cv. W29	JF920287	232,241	33 (3 rRNA, 17 tRNA)		
**Chloroplast genome**					
*B. rapa* cv. Chiifu-402-41, Z16 and FT	N/A	153,482	89 (4 rRNA, 37 tRNA)	Illumina Solexa sequencing, PCR, and Sanger sequencing	Wu et al., 2012
*B. rapa* cv. C1.3 (field mustard)	MG717286	153,483	N/A	Illumina HiSeq2500 sequencing	Ferreira de Carvalho et al., 2019
*B. rapa* cv. Z1 (field mustard)	MG717289	153,502			
*B. oleracea* cv. C1176	KR233156	153,366	80 (4 rRNA, 30 tRNA)	Illumina HiSeq short-read sequencing	Seol et al., 2017
*B. oleracea* cv. HDEM (wild cabbage)	MG717287	153,364	N/A	Illumina HiSeq2500 sequencing	Ferreira de Carvalho et al., 2019
*B. oleracea* cv. RC34 (wild cabbage)	MG717288	153,371			
*B. nigra* cv. IT119326	KT878383	153,633	80 (4 rRNA, 30 tRNA)	Illumina HiSeq short-read sequencing	Seol et al., 2017
*B. napus* cv. zy036	GQ861354	152,860	74 (4 rRNA, 30 tRNA)	Illumina Solexa sequencing, PCR and cycle sequencing	Hu Z.-Y. et al., 2011
*B. juncea*	KT581449	153,483	79 (4 rRNA, 30 tRNA)	Illumina HiSeq2500 sequencing, PCR, and Sanger sequencing	Prabhudas et al., 2016

Cp genomes of most land plants vary between 120 and 160 kbp in size (Wicke et al., 2011). Xiao-Ming et al. (2017) further established that cp gene lengths were proportionally to cp genome size, based on the analysis of 272 species including *B. napus* and 14 other members of the Brassicaceae family. Phylogenetic analysis of the cp genomes of *B. nigra* and *B. oleracea* with those of 10 reported species in the order Brassicales suggested that *B. oleracea* is closely related to *B. rapa* and *B. napus* while *B. nigra* was more diverse than the neighbor species *Raphanus sativus* (Seol et al., 2017). Li et al. (2017) completed the *de novo* assembly of cp genomes of 60 *Brassica* genotypes of the six U triangle species. Subsequent phylogenetic analyses divided the *Brassica* genus into four clades: *B. carinata* and *Brassica juncea*, in accordance with the U-triangle model, shared their cp genome with hybridization donors *B. nigra* and *B. rapa*, respectively. Two types of cp genomes were discovered in *B. rapa*, while the presence of both *B. rapa* cp genomes in *B. napus* strongly suggests two independent hybridization events. These findings were consistent with mt genome findings (Palmer and Herbon, 1988).

Besides photosynthesis, a number of essential metabolic reactions are catalyzed in the cp. These include the biosynthesis of partial amino acids, lipids and fatty acids, vitamins, and isoprenoids, as well as the reduction of nitrites and sulfates (Chen et al., 2018). The cp is furthermore involved in the synthesis defense-related hormones and signaling molecules metabolites associated with disease response and environment changes including heat and light (Lu and Yao, 2018). Genes present in the cp genomes could potentially be explored in *Brassica* to improve yield and resistance to biotic and abiotic stresses. It is clear that the composition and structure of the organellar genome not only hold potential in the elucidation of organellar genome evolution and phylogeny but and understanding thereof may be useful in the identification breeding compatible germplasm resources and CMS and provide opportunities for the introducing of new agronomic and horticultural traits into *Brassica* crops (Daniell et al., 2016). Organellar genome sequences are therefore valuable assets in the future crop improvement efforts.

## Functional Trait Discovery and Characterization

The characterization and subsequent exploration of discovered genetic diversity can uncover useful genes linked to adaptation and resistance to abiotic and biotic constraints (Khan et al., 2020). Available *Brassica* reference genomes have provided the foundation to facilitate the fine-scale mapping and elucidation of functionally significant variations in *Brassica* accessions. In this section, we review genome-wide approaches utilized in the identification of alleles associated with desirable traits.

### Genome-Wide Single-Nucleotide Polymorphism Discovery Through Whole Genome Re-sequencing

Available reference genomes provide a valuable tool in the study and detection of genetic variation, which can be reliably integrated and reproduced between studies (Malmberg et al., 2018). Alignment and comparison of WGRS data to the reference genome allow the simultaneous detection of large numbers of unbiased genomic single-nucleotide polymorphisms (SNPs), indels, and SVs. WGRS therefore permits a more in-depth interrogation of the genome than complexity reduction methods, resulting in a significant increase in the number of SNP markers detected.

Annotated, high-quality SNPs and SVs set the stage for high-resolution genome-wide association studies (GWASs). Quantitative trait locus (QTL) discovery can lead to the identification of genetic loci and subsequently provide the basis for the functional validation of candidate genes controlling important traits (Lu et al., 2019). Subsequently, by identifying these traits of interest, marker-assisted selection (MAS) could be introduced to advance breeding efforts for easily inherited traits (Harper et al., 2012). For example, in gene pyramiding, multiple genes or major QTLs are introgressed into a single genetic background (Pérez-de-Castro et al., 2012). This approach was recently implemented to introgress multiple genes conveying *Sclerotinia* resistance from wild *Brassica oleracea* into canola (Mei et al., 2020). Furthermore, as long-read sequencing technology advances and improves in accuracy, both PAVs and CNVs are likely to drive the latest source of rich adaptive variation including in crops subjected to biotic and abiotic stresses (Gabur et al., 2018).

In *Brassica* species, WGRS has facilitated the identification of intraspecific and interspecific genetic polymorphisms. Comparative analysis between different *Brassica rapa* morphotypes: Japanese turnip, rapid cycling, and Chinese cabbage cv. Chiifu, revealed, respectively 1,090, 1,118, and 1,464 unique genes in each of the genomes (Lin et al., 2014). Orthologous gene comparison suggested earlier divergence between the three varieties before a more recent domestication event. Gazave et al. (2016) used WGRS to survey the genetic diversity present in a worldwide collection of 782 accessions of *Brassica napus*. A total of 30,881 high-confidence SNP markers were identified, which upon analysis revealed distinct evolutionary histories for the A and C subgenomes. Wu et al. (2019) re-sequenced 991 spring, winter, and semi-winter *B. napus* germplasm accessions, originating from 39 countries. By mapping reads to the “Darmor-bzh” and “Tapidor” reference genomes, a total of 5.56 and 5.53 million SNPs, in addition to 1.86 and 1.92 million indels, were respectively identified. Comparison of SNPs using GWAS revealed a global pattern of genetic polymorphisms as well as paths of allelic drift within the main populations of *B. napus*. Selective sweeps disclosed the genetic basis of divergence between the ecotypes, while SNPs discovered in the promotor regions of FLOWERING LOCUS T and FLOWERING LOCUS C orthologs also corresponded with ecotype groups. Malmberg et al. (2018) furthermore utilized WGRS to develop genomic resources consisting of 4,029,750 high-confidence annotated SNPs with predicted effects, as well as SVs in the form of 10,976 deletions and 2,556 insertions. These valuable genomic resources have the potential to bring together global breeding efforts in the development of locally adapted *B. napus* varieties.

### Single-Nucleotide Polymorphism Arrays

Available sequencing data for *Brassica* crops allowed researchers to develop and use high-throughput molecular markers, such as SNPs, more efficiently (Clarke et al., 2013). These markers form an integral part of genomic diversity and, due to their abundance across the plant genome, have become an invaluable tool in crop improvement programs (Scheben et al., 2019). SNP screening can be carried out by WGRS, genotyping-by-sequencing (GBS), or alternatively using SNP arrays. High-density SNP arrays provide an alternative and reproducible genotyping platform, which has been widely used in the characterization of germplasm, GWAS, and QTL studies including the analysis of structural variation (You et al., 2018; Scheben et al., 2019).

A community-driven Brassica 60K (AC genomes) Illumina Infinium™ array (Clarke et al., 2016) was developed and more recently expanded to include the B-genome in the Brassica 90K Illumina Infinium™ array (Scheben et al., 2019). The usefulness of Brassica SNP arrays was demonstrated in the genotyping of resistance genes on chromosome A7 in *B. napus* (Dalton-Morgan et al., 2014), prediction of candidate genes for clubroot disease resistance in *B. napus* (Li et al., 2016), and the assessment of *de novo* homologous recombination events in *B. napus* (Higgins et al., 2018). In addition, several closely linked candidate genes were identified using the 60K Brassica SNP array in the development of functional haplotype markers for the improvement of the oleic acid content in rapeseed (Yao et al., 2020). Several yield associated traits have also been identified using the array platform including branching number (He et al., 2017), ovule numbers (Khan et al., 2019), seed quality (Gajardo et al., 2015), and stem strength (Li H. et al., 2018).

The creation of artificial *Brassica* allohexaploid could potentially result in the development of new oilseed and vegetable crops types with greater inter-subgenomic heterosis. These synthetic tri-genomic hexaploid *Brassica* species are potentially more vigorous and adaptable to a wider range of environmental conditions (Yan et al., 2009; Pradhan et al., 2010; Tian et al., 2010; Geng et al., 2013; Malek et al., 2013; Li et al., 2015; Gupta et al., 2016; Zhou et al., 2016; Mwathi et al., 2020). SNP genotyping was carried out by Gaebelein et al. (2019) using the 90K Brassica SNP array and GWAS to determine the relative impact of genome rearrangement events and inherited allelic variants on meiotic stability. A strong correlation between fertility and meiotic behavior in populations of *Brassica* allohexaploids segregating for alleles from parent allotetraploid species *B. napus, Brassica juncea*, and *Brassica carinata* was found. Potential genes of interest were subsequently identified for further investigation into meiotic regulation and future establishment of stable A, B, and C allohexaploids (Gaebelein et al., 2019).

Although SNP arrays can provide vital data to breeders and researchers, public accessibility to genotypes identified can be limited due to the lack of public repositories or databases designed to host crop SNP array data. To address this constraint, Scheben et al. (2019) established the CropSNP database (http://snpdb.appliedbioinformatics.com.au) for SNP array data generated on the Illumina Infinium™ Brassica 60 and 90K array platforms.

### Transcriptomics

One of the key challenges in genomics-based breeding remains the complex linking of genotype to phenotype across tissue types, developmental stages, and environmental conditions. Transcriptomics, although part of associated “omics,” have emerged as an exceptional tool in the functional inference of genetic variability (Wang et al., 2019) with technological innovation constantly advancing the field (Lowe et al., 2017; Wang et al., 2019).

RNA-Seq technology has been extensively used in the mapping of exon/intron boundaries, improvement of genome annotations, and the detection of rare transcripts and splicing variants (Pérez-de-Castro et al., 2012). For example, the transcriptional regulation of anthocyanin biosynthesis in *B. juncea* was studied to identify differentially expressed genes between the purple and green leaves from a backcrossed BC3 segregation population. Genes associated with phenylpropanoid biosynthesis, phenylalanine metabolism, and flavonoid biosynthesis were differentially expressed, while genes involved with anthocyanin biosynthesis (*BjTT8* and *BjMYC2*) were up-regulated in the purple leaves. Understanding anthocyanin biosynthesis and its regulatory network in *Brassica* is a prerequisite in the development of health-promoting anthocyanin-rich vegetables (Heng et al., 2020).

The complex defense response between a *Sclerotinia sclerotiorum* resistant and susceptible line of *B. napus* was analyzed in a study by Wu et al. (2016). Dynamic transcriptome analyses uncovered differences between susceptibility and resistance associated with the magnitude of expression changes in genes involved in pathogen recognition, MAPK signaling cascade, WRKY transcription regulation, jasmonic acid/ethylene signaling pathways, and biosynthesis of defense-related protein and indolic glucosinolate. Valuable insights gained will assist in the development of effective strategies in *Sclerotinia*-resistance breeding (Lu et al., 2014; Wu et al., 2016).

Limitations such as misassembly, associated with short read RNA-Seq, hampers the full-length assembly of transcripts from highly repetitive regions or analogous gene families. These difficulties are often more pronounced in polyploid plants. TGS technologies such as SMRT by PacBio and Oxford Nanopore single molecule structure sequencing (SMS-seq) provide an opportunity to construct full-length transcripts, with the possibility to capture structural variations, tertiary interactions, and the dynamics of riboswitch ligand binding (Bizuayehu et al., 2020). Full-length transcriptome sequencing (PacBio) was used by Tan et al. (2019) to explore the transcript and splice isoforms expressed during anther development in Chinese cabbage (*B. rapa* ssp. *pekinensis*). In addition to predicted fusion transcripts and poly-A sites, 53 key genes active during anther development were detected, of which eight annotated loci had alternatively spliced isoforms. The transcripts generated provided a valuable resource for the characterization of anther-specific gene expression and improved Chinese cabbage genome annotation (Tan et al., 2019).

An et al. (2019) conducted a comprehensive study comparing the genetic diversity of 183 *B. napus*, 112 *B. rapa*, and 42 *B. oleracea* accessions, along with 20 wild relatives (*Brassica hilarionis, Brassica villosa, Brassica montana, Brassica macrocarpa, Brassica rupestris, Brassica incana*, and *Brassica insularis*) and five other Brassicaceae species as outgroups in order to improve the understanding in the origin and diversification of *B. napus*. RNA-Seq reads generated from *B. rapa* accessions and *B. oleracea* and other wild C genome species were respectively mapped to the A and C genomes of the *B. napus* Darmor-bzh reference genome to identify SNPs. Six genetic clusters of *B. napus* were identified, which were shown to have undergone different selective pressures in accordance with known breeding histories. Although the multi-origin of *B. napus* remained elusive, the study contributed toward the identification of putative candidate genes to important agronomic traits, which, along with high-quality SNPs identified, have the potential to facilitate rapeseed improvement and germplasm preservation.

In line with the pangenomics approach, He et al. (2015) assembled the first pan-transcriptome resources for the *Brassica* A and C genomes. The pan transcriptome was established using existing coding DNA sequence (CDS) gene models from the *B. oleracea* TO1000 and *B. napus* Darmor-bzh reference genomes in addition to preliminary CDS models from the *B. rapa* Chiifu genome sequence assembly. The construction of the pan-transcriptome allows, in a similar fashion as the pangenome, the discovery of functional dispensable genes (Jin et al., 2016).

### Epi-Genomics

In addition to the identification of novel genes and useful haplotypes, epigenetic variation has the potential to contribute toward crop adaptation and productivity (Dwivedi et al., 2017). These epigenetic variations are plant developmental and adaptation responses to environmental constraints (Gallusci et al., 2017; Tirnaz and Batley, 2019b). Epigenomics encapsulates genotype × environment interactions and their independent influence on the phenotype (Seymour and Becker, 2017) and may contribute as potential phenotypic resources for breeding.

Epigenetic regulation is independent of DNA sequence alteration and stably inherited during mitosis or meiosis (Weigel and Colot, 2012). DNA (de)methylation, histone modification, and chromatin remodeling involves regulatory reprogramming at transcriptional and post-transcriptional levels (Paszkowski and Whitham, 2001; Tirnaz and Batley, 2019a). DNA methylation in plants includes *de novo* methylation, as well as maintenance and demethylation, as a means of regulatory check and balance in gene expression (Elhamamsy, 2016; Tirnaz and Batley, 2019a). Histone modification refers to the methylation or (de)acetylation of histone proteins at the N-terminal. Mono-, di-, or trimethylation of the lysine residue in the former results in various functional responses, while the histone acetylation and deacetylation are associated with gene activation and repression, respectively (Fuchs et al., 2006). Additionally, chromatin remodeling is influenced by histone octomer movement (Perrella and Kaiserli, 2016), ATP-dependent enzyme affecting nucleosome composition (Tariq and Paszkowski, 2004) or histone variants (Rando and Ahmad, 2007) cause the DNA sequence to become inaccessible to transcriptional mechanisms, resulting in transcriptional silencing.

Epialleles, or epivariants, first coined in mammals by Rakyan et al. (2002), refer to genetically identical and stable alleles that are variably expressed due to epigenetic regulations and result in difference in phenotype (Richards, 2006; Dolinoy et al., 2010). Earlier studies on epialleles lack whole-genome information to decipher association with desirable traits (Seymour and Becker, 2017). While more studies have been conducted recently, epigenomics is still considered in its infancy compared with the characterization of genetic sequence- or structural-caused variation.

Epigenomic studies in *Brassica* crops have predominantly involved global or targeted methylation profiling for natural or experimentally induced epivariants as defined by Gallusci et al. (2017). Early *Brassica* methylation studies relied on a chemical demethylation agent treatment using 5-azacytidine (5-AzaC) and on cytological (Solís et al., 2015) as well as targeted allele-specific and methylation-sensitive amplified polymorphism (MSAP) techniques to capture the global DNA methylation pattern (Shiba et al., 2006; Hauben et al., 2009) (refer Table 3). Hypomethylated populations created using 5-AzaC treatment could also be mined for epiallelic variation (Amoah et al., 2012). The forward and reverse screening of epigenetic variation was employed in functional and inheritance studies in *B. rapa* var. *trilocularis* and suggested to have potential as an intervention strategy for crop improvement (Amoah et al., 2012).

**Table 3:** Exploration of epigenetic resources in *Brassica* for natural and experimentally induced epivariants.

**Approach**	***Brassica* species**	**Trait of interest**	**Source of epigenetic variation**	**References**
Allele-specific bisulfite sequencing	*Brassica rapa*	Self-incompatibility	Natural	Shiba et al., 2006
Methylation-sensitive AFLP, cytosine extension assay	*Brassica napus*	Energy use efficiency	Experimental	Hauben et al., 2009
MSAP	*B. rapa* cv. *Osome*	Chromatin remodeling	Experimental	Sasaki et al., 2011
MSAP	*B. rapa* var. *trilocularis* cv. R-o-18	Hypomethylated epialleles	Experimental	Amoah et al., 2012
WGBS using NGS	*B. napus* var. *oleifera*	Salt stress	Natural	Marconi et al., 2013
WGBS using NGS	*Brassica oleracea* cv. TO1000	Cystosine methylation	Natural	Parkin et al., 2014
Reduced representation bisulfite sequencing (RBBS)	*B. rapa* var. *oleifera* cv. 3H120	Hybridization and polyploidy	Natural	Chen et al., 2015
ChIP	*B. rapa* var. *pekinensis* cv. RJKB-T23 and RJKB-T24	Vernalization, high bolting	Natural	Kawanabe et al., 2016
MSAP	*B. oleracea* var. *botrytis* cv. Korso × *Brassica nigra* cv. G1/1 introgression lines	Methylation pattern and heredity	Natural	Wang G.-X. et al., 2016
WGBS using NGS	*B. oleracea* cv. 01-20S and 01-20F	Male sterility	Natural	Han et al., 2017
Methylated DNA immunoprecipitation sequencing (MeDIP-seq)	*B. rapa* ssp. *chinensis* cv. NHCC004	Heat tolerance	Experimental	Liu T. et al., 2017
WGBS using NGS	*B. rapa* cv. Suzhouqing (NHCC001)	Heat stress	Natural	Liu G. et al., 2018
Methylated DNA immunoprecipitation sequencing (MeDIP-seq)	*B. rapa* cv. RJKB-T23 and RJKB-T24	Fusarium disease resistance	Natural	Takahashi et al., 2018a
WGBS using NGS, small RNA-Seq, and ChIP	*B. rapa* var. *pekinensis* cv. RJKB-T24	DNA methylation and expression, and H3K9me2 modification and small RNA expression	Natural	Takahashi et al., 2018b
WGBS using NGS	*B. napus* cv. 7365A and 7365B	Male sterility	Natural	Wang Z. et al., 2018
ChIP sequencing (ChIP-seq) and RNA-Seq	*B. rapa* var. *pekinensis* RJKB-T23 and RJKB-T24 inbred lines	H3K27me3 in *FLC* regulation and vernalization	Natural	Akter et al., 2019
ChIP sequencing (ChIP-seq) and RNA-Seq	*B. rapa* ssp. *trilocularis* cv. R-o-18	H3K27me3 in epigenetic regulation	Natural	Payá-Milans et al., 2019
ChIP sequencing (ChIP-seq) and RNA-Seq	*B. rapa* ssp. *chinensis* cv. *Aijiaohuang*	Pollen wall formation	Experimental	Shen et al., 2019
Targeted enrichment using seq-cap epi	*B. napus* cv. Westar and Sturt	Resistant to *Leptosphaeria maculans*	Natural	Tirnaz et al., 2020
WGBS using ONT	*B. nigra* cv. C2 and Ni100	Active centromeres	Natural	Perumal et al., 2020

*AFLP, amplification fragment length polymorphism; MSAP, methylation-sensitive amplified polymorphism; WGBS, whole-genome bisulfite sequencing; NGS, next-generation sequencing; ChIP, chromatin immunoprecipitation; ONT, Oxford Nanopore Technologies*.

These techniques were superseded by whole-genome bisulfite sequencing (WGBS) first described in *Arabidopsis* (Cokus et al., 2008; Lister et al., 2008). With the use of the WGBS approach, DNA hypomethylation of the multiallelic *Bnams4* gene associated with male sterility was detected in young floral buds (Wang Z. et al., 2018). The male sterility trait promotes heterosis and hybrid development and hence is favored in crop improvement and breeding strategies (Saxena and Hingane, 2015).

Chromatin immunoprecipitation (ChIP) is another useful strategy for protein–gene interaction studies and, in the case of methylation, for investigating specific histone modifications (Das et al., 2004; Kawanabe et al., 2016). Kawanabe et al. (2016) developed positive and negative control primers to validate ChIP assays. The primers were targeted at histone modifications at H3K4me3 (trimethylation of the 4th lysine of H3), H3K9me2, H3K27me3, and H3K36me3 and used to study the response of 4 *FLC* paralogs to vernalization in *B. rapa* var. *pekinensis*. ChIP combined with WGBS further expanded the ability to investigate complex interactions between genetic and epigenetic factors (Li and Tollefsbol, 2011). These studies assisted in the identification of epigenetic markers, epigenetic QTL, and genes associated with floral and pollen development, self-incompatibility, salt and heat stress, vernalization, disease resistance, and male sterility and in the assessment of methylation profiles of introgression lines for crop improvement resource (Table 3). Nonetheless, due to the complexity of epigenetic interaction and its involvement in complex regulatory networks, these epigenomic approaches still require extensive investigation before their application in crop improvement can be implemented.

### Reverse Genetics

One of the most cost-effective and quick approaches used to identify genetic variation in crop populations is by Targeting Induced Local Lesions in Genomes (TILLING). This method combines chemical mutagenesis, to create lesions on the genome, and molecular techniques such as PCR and DNA pooling, to identify point mutations within the population. TILLING was first demonstrated in *Arabidopsis* using ethyl methanesulfonate (EMS) (McCallum et al., 2000), which results in base transitions by causing the G residues to alkylate and as a result pair with T instead of C (Rashid et al., 2011). Soon after, the technique was explored in *Brassica* species for various traits of interest including fatty acid content and shatter tolerance (Table 4). However, this method is less feasible and becomes more time-consuming if multiple genes are targeted, such as those involved in specific biopathways (Sashidhar et al., 2020). Examples of canola varieties developed that benefit from this approach include PodGuard trait for In Vigor R5520P and 1H51 RR varieties, which were commercially marketed by Bayer for their resistance to pod shattering (Raman et al., 2019).

**Table 4:** Reverse genetic resource in *Brassica* over the last 10 years.

**Approach/Species**	**Trait of interest (target gene(s))**	**References**
**RNAi**		
*B. rapa* cv. Osome	Self-compatibility S-locus (*SRK, SP11/SCR*)	Jung et al., 2012
*B. juncea* cv. Varuna	Glucosinolate transcriptional regulator (*MYB28*)	Augustine et al., 2013
**TILLING**		
*B. napus* cv. Ningyou 7	Seed erucic acid content (*FAE1*)	Wang et al., 2008
*B. napus* cv. YN01-429 x Express 617	Sinapine synthesis (*SGT, REF1*)	Harloff et al., 2012
*B. napus* cv. DH12075	Demonstrate mutation density (39 genes)	Gilchrist et al., 2013
*B. napus* cv. Express 617	Phytic acid (six gene families: *MIPS, MIK, 2-PGK, IPK1, IPK2*, and *MRP5*)	Sashidhar et al., 2019
Three *B. rapa* ssp. *trilocularis* cv. R-o-18 mutants	CAX1 transporter for Calcium homeostasis (*BraA.cax1a*)	Navarro-León et al., 2018
*B. oleracea* cv. TO1000	Abiotic stress response (15 genes)	Himelblau et al., 2009
*B. rapa* ssp. *trilocularis* cv. R-o-18	Redundant genes function (three *REPLUMLESS*, one *INDEHISCENT*, two *METHYLTRANSFERASE1*)	Stephenson et al., 2010
**EcoTILLING**		
101 *B. napus*, nine *B. oleracea* and seven *B. rapa* accessions	Seed erucic acid content (*FAE1*)	Wang et al., 2010
**ORG-EcoTILLING**		
90 *B. napus*, three *B. rapa*, and three *B. oleracea* accessions	Breeding history and maternal inheritance (*accD, matK, rbcL*)	Zeng et al., 2012
**VIGS**		
*B. napus* (winter varieties)	Flowering (*FLC*)	Álvarez-Venegas et al., 2011
*B. rapa* ssp. *Chinensis* cv. Wuyueman and 49 Caixin	Flowering (*MAF*)	Huang et al., 2018
*B. rapa* cv. Sijiucaixin	Carotenoid biosynthetic pathway (*PDS*)	Yu et al., 2018

TILLING has been further developed to include EcoTILLING (Ecotype) and ORG-EcoTILLING. EcoTILLING involves pooling DNA from only two individuals consisting of a reference and queried genotypes (Backes, 2013); therefore, instead of creating mutant populations, this more recent approach involves a study of allelic variation. EcoTILLING requires re-sequencing efforts to characterize and locate the genotypic polymorphism (Wang et al., 2010). Additionally, this approach is high-throughput and can associate natural variants with gene function, trait association, and phylogenetic relationships (Zeng et al., 2012). ORG-EcoTILLING was first utilized in Brassicas to explore the use of TILLING in organelle genomes by combining CEL1 endonuclease, which cuts specific mismatches in heteroplex DNA, and PCR, for three cp genes and one mt gene. ORG-EcoTILLING consistently confirmed *B. rapa* as *B. napus* maternal progenitors based on phylogenetic analysis. Additionally, it also uncovered the possibility of multiple origins and evolution throughout *B. napus* domestication with the identification of three additional divergences in the accessions.

The TILLING approach is considerably more accessible in terms of cost and time than other specialized reverse genetics approaches like RNA interference (RNAi) or gene silencing, and virus-induced gene silencing (VIGS). RNAi gained traction with the ability to knock down gene functions mediated by small interfering RNA (siRNA) or microRNA (miRNA) (Pe'ery et al., 2003; Limera et al., 2017). Traits introduced using RNAi, such as self-compatibility in *B. rapa*, have been found to be stable even in crosses with commercial variety (Jung et al., 2012). This will help improve likelihood for the seeds to be used in commercial cultivation. Similar to RNAi, VIGS also involves introduction of dsRNA molecules. A viral vector genome such as *Cabbage Leaf Curl Virus* (CaLCuV) is modified to include the plant target gene fragment (150–800 bp) and to remove the viral inducing host gene, thus forming a recombinant virus (Lu et al., 2003; Ramegowda et al., 2014; Bekele et al., 2019). The recombinant vector introduces infection in the plant, is amplified, and generates dsRNA molecules. These dsRNA molecules triggered post transcriptional gene silencing, once detected by the host plant, causing it to be cleaved into siRNA. The RNAi silencing complex and antisense siRNA strands associates together and begin to target RNAs, which complemented the siRNAs. These target-specific RNAs were screened and destroyed, which subsequently caused the target gene to be silenced. VIGS can also be utilized for tissue-specific gene silencing, helpful in screening stress responses, and induced transcriptional gene silencing by targeting the gene promoter (Kanazawa et al., 2011; Senthil-Kumar and Mysore, 2011; Bekele et al., 2019). VIGS research in *Brassica* includes interest in understanding the vernalization pathway in Brassicas as a means to improve flowering and reproductive development by silencing genes associated with late flowering (Álvarez-Venegas et al., 2011) or floral organ transition (Huang et al., 2018).

## Genome Manipulation

Traditional breeding approaches rely on the diversity found in local land races, mutation panels, or even germplasm from related species to introduce desired traits or elite alleles through costly and time-consuming backcrossing programs (Dwivedi et al., 2017). The absence of natural genetic diversity and potential linkage drag introducing closely linked unwanted agronomic characteristics has plagued crop improvement efforts (Holme et al., 2013). Increased availability of genomic resources, identified and well-characterized genes, as well as a deeper understanding of underlying molecular mechanisms has led the way for the introduction of innovative approaches to overcome these limitations and fast-track crop breeding (Scheben et al., 2016: Hickey et al., 2019).

### Gene Transformation

Genetic modification through the introduction of transgenes was developed in an attempt to expand the available genepool (Kamthan et al., 2016). *Agrobacterium tumefaciens* and biolistic techniques are widely and efficiently employed to mediate the transfer of selected exogenous genes or regulatory elements from an unrelated species or even non-plant organism (Moloney et al., 1989; Altpeter et al., 2016). Numerous transgenic *Brassica* spp. have been developed in an attempt to introduce traits such as salt tolerance (Kim et al., 2016), disease resistance (Grison et al., 1996; Aghazadeh et al., 2016; Zarinpanjeh et al., 2016), reduced sinapine content (Wolfram et al., 2010; Harloff et al., 2012), and herbicide tolerance (Beversdorf et al., 1988; De Block et al., 1989; Cuthbert et al., 2001). The potential of transgenics in advanced plant metabolic engineering is however best demonstrated in the development of transgenic omega-3 *Brassica napus* varieties by BASF and Cargill, and Nuseed, CSIRO and GRDC (Napier et al., 2019) respectively, as well as *Brassica juncea* by Wu et al. (2005). Transgenic *B. napus* accumulating long-chain polyunsaturated fatty acids (LC-PUFAs) were engineered by each of the initiatives through the introduction of large multi-transgene cassettes. The BASF cassette (~44 kbp) contained 12 genes, while the Nuseed initiative's cassette (~23 kbp) contained six omega-3 LC-PUFA biosynthetic genes. Each gene was under the regulation of a seed-specific promoter. Each cassette also contained a gene for herbicide resistance (Connelly and MacIntosh, 2018; Sottosanto et al., 2018). The LC-PUFA profile produced by the two omega-3 transgenic canola varieties varied and respectively contained ~7% eicosapentaenoic acid (EPA), ~3% docosapentaenoic acid (DPA), and ~1% docosahexaenoic acid (DHA) (LBFLFK); and <0.5% EPA, ~1% DPA, and ~10% DHA (NS-B50027-4) respectively (Napier et al., 2019). Very-long-chain (VLC) PUFA accumulating *B. juncea* was on the other hand engineered in a stepwise approach through a series of transformations with increasing numbers of transgenes. The resulting transgenic *B. juncea* yielded up to ~15% EPA, ~4% DPA, and ~1.5% DHA (Wu et al., 2005).

In addition to the transformation of the nuclear genome, modification of the cp genome has also been established. The introduction of foreign genes into the cp can address nuclear transgenic limitations such as low-level transgene expression (Jin and Daniell, 2015) and the potential transgene escape via pollen (Daniell, 2007). The stable integration and expression of more than 40 cp-based transgenes has been reported by Daniell et al. (2016), most of which were aimed at potentially enhancing biotic stress tolerance and consequently yield. An example is the development of a cabbage-plastid transformation system for introduction of the insecticidal *cry1Ab* gene. With the use of a species-specific vector, the expression of the BT-toxin facilitated the control of the diamond moth, an economically important *Brassica* pest (Liu C. W. et al., 2008). Introduced insect resistance using *cry* genes was also reported for rapeseed (Schuler et al., 2004) and collards (Cao et al., 2005).

Improved weed and insect control using GM crops has assisted farmers in attaining higher yields with fewer resources. The analysis by Brookes and Barfoot (2018) estimated that the adoption of commercial herbicide-tolerant GM canola and sugar beet led to a total global income gain of $559 million in 2016 and $6.44 billion cumulatively since 1996. Despite the commercial benefits, growing consumer skepticism (Frewer et al., 2013), as well as potential risks to human health and the environment (Zhang et al., 2016), has encouraged the development of alternative genomic modification technologies.

Cisgenesis and intragenesis, based on the same gene transfer technologies as transgenesis, were consequently developed. Genetic crop modification thereby involved the introduction of target DNA from the same plant species, or a sexually compatible species, for crop improvement. Resulting crop plants are free from any foreign DNA mitigating associated risk to some extent (Espinoza et al., 2013; Holme et al., 2013). Alternative approaches such as reverse breeding exploit the use of transgenes to accelerate initial breeding. The unwanted transgene is eliminated through Mendelian segregation during the later stages of the breeding process (Dirks et al., 2009; Basso et al., 2020).

### Genome Editing

Recently, GE technologies have come to the foreground, allowing the precise and permanent modification of specific genes or genomic regions. ODM, programmable SSNs, and base editing provide an opportunity to study gene function and alter crop traits through the mutation of specific genes, reprogramming of epigenetic markers, and the generation of site-specific sequence modifications (Voytas and Gao, 2014; Ran et al., 2017; Jansing et al., 2019).

#### Oligo-Directed Mutagenesis

Traditional mutation breeding using chemical or irradiation results in random mutations in the genome. The movement toward a more desired and controlled site-specific targeted mutagenesis took shape in the 1970s (Lusser and Davies, 2013). ODM, also known as targeted gene repair, oligonucleotide-directed gene targeting, genoplasty, and chimeraplasty, makes use of 20–100 bp of DNA or RNA oligonucleotides to introduce mutations at the target site. The synthesized oligonucleotides are designed to be homologous to the target site with the exception of 1–4 bp (Lusser et al., 2012). Upon transfection, the oligonucleotides associate with the target site, prompting DNA repair at the sequence mismatch sites resulting in base pair mutations, deletions, or reversal of mutations (Lusser et al., 2012; Lusser and Davies, 2013). With a difference of often just a few nucleotides underlying important traits in plants, the application of ODM held potential as a non-GM organism (GMO) base pair-specific oligonucleotide-directed gene editing platform to augment the genetic diversity of a specific genotype. The use of ODM was furthered by Cibus as part of the commercial Rapid Trait Development System (RTDS™) introducing novel and commercially valuable traits such as herbicide resistance into a variety of crops including oilseed rape (Gocal et al., 2015). Ruiter et al. (2003) found that spontaneous mutation in plants obscured the intended sequence modifications in *B. napus* mediated through self-complementary RNA–DNA chimeric oligonucleotides or chimeraplasty. Studies by Sauer et al. (2016) confirmed that significant precise gene-editing events in plants could be realized by ODM alone and suggested that ODM efficiency could be further improved in combination with reagents that cause DNA double-stranded breaks (DSBs).

#### Programmable Sequence-Specific Nuclease

GE using programmable SSNs is generally achieved through the induction of a controlled DSB at a target locus using SSNs. The DBS activates the intracellular DNA-repair pathways and is repaired through either non-homologous end joining (NHEJ) or homology-directed repair (HDR) (Gaj et al., 2013). The imprecise re-joining of the DBS through NHEJ leads to the introduction of indels at the target loci and disruption of gene function. On the contrary, HDR entails the use of an exogenous DNA-repair template to bridge the DSB site. The repair template, a double-stranded DNA vector or a single-stranded DNA oligonucleotide, enables the introduction of a precise mutation or insertion to alter gene function (Zhang et al., 2013). Several engineered nuclease systems have been developed including meganucleases (MN), zinc finger nucleases (ZFNs), transcription activator-like effector nucleases (TALENs), and clustered regularly interspaced short palindromic repeats (CRISPR) coupled with a CRISPR-associated protein (Cas).

##### Zinc Finger Nucleases

ZFN, first described by Kim et al. (1996), employs novel hybrid site-specific endonucleases created by the linking of two different zinc finger proteins (ZFPs) to the cleavage domain of the bacterial FokI endonuclease. The zinc finger domains are designed to each recognize and bind to a unique 3- to 4-bp DNA sequence adjacent to the target site. The tandem repeats can be constructed to recognize an extended 9- to 18-bp DNA sequence (Lin and Musunuru, 2016). In an attempt to modify seed oil composition in *B. napus*, Gupta et al. (2012) engineered ZFP transcription factors (TFs) to fuse to a conserved region downstream of the transcription start site of two canola KASII genes. The modification resulted in the escalated expression of the KASII mRNA, increasing C18 and lowering palmitic acid levels well as the overall saturated fatty acid content in the seed. Canola oil with a lower saturated fat content is more desirable and potentially poses health benefits (Hyseni et al., 2017). Despite the progress in technology, the engineering of the desired ZFN-binding domain remains challenging and time-consuming, with further limitations presenting in selection of the target site (Cox et al., 2015).

##### Transcription Activator-Like Effector Nucleases

TALENs are similar in structure to ZFNs, composed of di-meric DNA-binding proteins fused to the nuclease domain FokI (Cermak et al., 2011). The central domain of the TAL effector consists of a polymorphic repeat of ~34 amino acids with hypervariable di-amino acids at positions 12 and 13. These so-called repeat variable di-residues (RVDs) associate and recognize a corresponding C, T, A, or G nucleotide (Scholze and Boch, 2010). The longer DNA recognition sites promote specificity and reduce potential off-target effects (Li et al., 2012). The TAL effector DNA binding domain is more flexible and can be customized, which broadens its potential application.

Sun et al. (2013) demonstrated the suitability of the TALEN construct to alter the endogenous FRIGIDA (vernalization determinant) gene in *Brassica oleracea* var. *capitata*, further suggesting that the method could be applied to related *Brassica* spp. TALENs with mt localization signals (mitoTALENs) were designed by Kazama et al. (2019) to knock out CMS-associated genes at orf79 and orf125, respectively, of CMS varieties of rice and *B. napus* (SW18). Induced mt modifications restored fertility without causing noticeable phenotypic changes and were found to be stable and maternally inherited. The successful modifications of the mt genome pose the prospect of “mitochondrial breeding” in plants (Kazama et al., 2019), which can play an important role in the study and future conditioning of plant responses toward climate change (Budar and Roux, 2011; Sweetman et al., 2019; Florez-Sarasa et al., 2020). TALENs similar to ZFNs are time-consuming genome manipulation techniques (Razzaq et al., 2019) and require the extensive screening of large numbers of manipulated individuals.

##### RNA-Guided Nucleases

The second-generation CRISPR/Cas9 system provides an alternative approach in targeted nucleases. In contrast to the ZFN's and TALEN's engineered protein associated DNA-binding systems, the CRISPR/Cas9 system has a single-guide RNA (sgRNA) bound to the Cas9 endonuclease that directs the complex to a specific site in the genome (Jinek et al., 2012; Cong et al., 2013). Recognition is attained through base pairing between the programmable 20-bp-long spacer region at the 5′-end leading sequence of the gRNA and specific DNA target. The Cas9 nuclease uses the CRISPR gRNA–DNA pairing as guide in combination with adjacent the DNA protospacer-adjacent motif (PAM) to cleave the DNA. This simplicity and flexibility in the programming of the CRISPR/Cas9 system have facilitated its adoption and exploitation in plants including *Brassica* (Zhang et al., 2018b). Lawrenson et al. (2015) demonstrated the efficiency of the CRISPR/Cas9 GE tool for the first time in the knockout of target genes in *B. oleracea*. Introduced mutations were stably inherited and transgene-free plants obtained through segregation.

Due to the allotetraploid nature of some *Brassica* species, the observed effect of single gene modification is often limited by its potential redundant function. Modification of all homologous genes is hence required to obtain a reliable altered genotype and phenotype (Sashidhar et al., 2019). Multiple guide sequences can be encoded into a single CRISPR array, allowing the simultaneous editing of several sites (Cong et al., 2013), making the CRISPR/Cas9 system a valuable tool in the knockout of redundant genes or parallel pathways in polyploids. Sashidhar et al. (2019) illustrate a case in point with the CRISPR/Cas9-mediated knockout of multiple paralogs of the key enzyme inositol tetrakisphosphate kinase (ITPK) involved in the synthesis of phytic acid in *B. napus* seed. A noticeable change in the phytic acid content of *B. napus* seed was observed only in triple mutants of the essential *BnITPK* genes.

The CRISPR/Cas9 GE tool has been widely adopted in the manipulation and study of a variety of genes underlying agronomical important traits in *Brassica*. These include traits such as pod shatter resistance (Braatz et al., 2017; Zhai et al., 2019), multi-ocular silique (Yang Y. et al., 2018), increase in oleic acid content in seed (Okuzaki et al., 2018), and seed coat color (Zhai et al., 2020) (Table 5). Even though the use of the CRISPR/Cas9 system is popular in gene knockout or knock-in studies, it is limited by the introduction of random indels at the target site in addition to the possibility off-target mutations.

**Table 5:** Application of programmable sequence-specific nucleases and targeted base editing tools in genome editing of *Brassica*.

***Brassica* species**	**Target gene/s and modification**	**Editing system**	**Modified/introduced trait**	**References**
**Programmable sequence-specific nucleases**				
*Brassica napus*	Introduction of ZFP-transcriptional activators (TF) to activate two canola β-ketoacyl-ACP Synthase II designed (*KASII*) genes	ZFN	Elevated KASII transcript levels, decrease in palmitic acid, increased in total C18, and reduced total saturated fatty acid content	Gupta et al., 2012
*Brassica oleracea var. capitata*	Targeted cleaving of the endogenous *FRIGIDA* (*FRI*) gene	TALENs	Earlier flowering	Sun et al., 2013
*B. napus*	Knockout of CMS-associated genes (*orf125*)	mitoTALENs	Restored male fertility	Kazama et al., 2019
*B. oleracea*	Indel mutations of the *BolC.GA4a*	CRISPR/Cas9	Dwarf phenotype and alterations in pod valve margins	Lawrenson et al., 2015
*B. napus*	Knockout mutations of *BnaRGA, BnaDA2*, and *BnaFUL*	CRISPR/Cas9	Demonstrate CRISPR/Cas9 efficiency in creating targeted genome modifications at multiple loci that are stable and inheritable	Yang et al., 2017
*B. napus*	Induced mutation in *BnWRKY11* and *BnWRKY70*	CRISPR/Cas9	*BnWRKY70* mutants exhibited enhanced resistance to *Sclerotinia*, while *BnWRKY11* mutants showed no significant difference in *Sclerotinia* resistance	Sun et al., 2018
*B. napus*	Modification of a fatty acid desaturase 2 gene (*FAD2*)	CRISPR/Cas9	Increase of oleic acid content seed	Okuzaki et al., 2018
*B. napus*	Knockout of rapeseed homologs of *CLAVATA3* (*CLV3*)	CRISPR/Cas9	Produced more leaves, multilocular siliques, and higher number of seed	Yang Y. et al., 2018
*B. napus*	Knockout of *INDEHISCENT* (*IND*) and *ALCATRAZ* (*ALC*) gene homologs	CRISPR/Cas9	Association of the *BnIND* gene with pod shatter resistance, whereas the *BnALC* gene appears to have limited potential for rapeseed shatter resistance	Tang et al., 2018
*B. napus*	Knockout of SPL3 homologous gene copies, *BnSPL3*-*A5/BnSPL3-A4/BnSPL3-C3/BnSPL3-C4/BnSPL3-Cnn*	CRISPR/Cas9	Developmental delay	Li C. et al., 2018
*B. napus*	Knockout mutations of the histone lysine methyltransferases *BnaSDG8.A* and *BnaSDG8.C*	CRISPR/Cas9	*BnaSDG8.A* and *BnaSDG8.C* help control the *B*. *napus* floral transition by directly altering the H3K36m2/3 levels at the *BnaFLC* chromatin loci	Jiang et al., 2018
*B. oleracea* var. *capitata*	Induced mutations in phytoene desaturase gene *BoPDS*, S-receptor kinase gene *BoSRK*, male-sterility-associated gene *BoMS1*	CRISPR/Cas9 based on endogenous tRNA processing	*BoSRK3* gene mutation suppressed self-incompatibility completely *BoMS1* gene mutation produced a completely male-sterile mutant	Ma et al., 2019
*B. napus*	Induced mutation in *INDEHISCENT* (*IND*) and *ALCATRAZ* (*ALC*)	CRISPR/Cas9	Establishes that *BnIND* gene is essential for pod shatter and highly conserved in *Brassica*	Zhai et al., 2019
*B. napus*	Knockout of seven *BnLPAT2* homologous genes and four *BnLPAT5* homologous genes	CRISPR/Cas9	Enlarged oil bodies, disrupted distribution of protein bodies and increased accumulation of starch confirming the role of *BnLPAT2* and *BnLPAT5* in oil biosynthesis	Zhang et al., 2019
*B. napus*	Knockout of five homoeologs (*BnJAG.A02, BnJAG.C02, BnJAG.C06, BnJAG.A07*, and *BnJAG.A08*) of the *JAGGED* (*JAG*) gene	CRISPR/Cas9	Affected development of the lateral organs in organizing pod shape and size as well as viable seed. *BnJAG.A08-NUB* gene caused significant changes in the pod dehiscence zone	Zaman et al., 2019
*B. napus*	Knockout mutations of *BnaMAX1* homologs	CRISPR/Cas9	Semi-dwarf and increased branching phenotypes with more siliques, contributing to increased yield per plant.	Zheng et al., 2020
*B. napus*	Mutation of the M-locus protein kinase (MLPK) *BnaMLPK* genes	CRISPR/Cas9	Revealed that MLPK is a positive regulator of the self-incompatibility response	Chen et al., 2019
*B. napus*	Knockout mutations of the *BnA10.LMI1* gene	CRISPR/Cas9	Demonstrated *BnLLA10* regulates the development of leaf lobes	Hu et al., 2018
*Brassica carinata*	Mutation of the *FASCICLIN-LIKE ARABINOGALACTAN PROTEIN 1* (*BcFLA1*) gene	CRISPR/Cas9	Down regulation of *BcFLA1* decreased root growth under Pi-deficient conditions predicting role	Kirchner et al., 2018
*B. napus*	Knockout mutation of the *APETALA2* (*BnAP2*) genes	CRISPR/Cas9	Loss of *AP2* function severely affects sepal and petal development and generates sepal carpeloid and apetalous mutants	Zhang et al., 2018a
*Brassica campestris*	Induced *BcPME37c* mutant	CRISPR/Cas9	*BcPME37c* mutation led to the abnormal thickening of the pollen intine	Xiong et al., 2019
*B. napus*	Targeted knockout of *TRANSPARENT TESTA 2* (*BnTT2*) homologs	CRISPR/Cas9	*BnTT2* homologs had conserved but redundant functions in regulating seed color. Homozygous mutants of *BnTT2* homologs increased oil content and improved fatty acid composition with higher linoleic acid (C18:2) and linolenic acid (C18:3)	Xie et al., 2020
*B. napus*	Knockout lines of *BnaA9.WRKY47*	CRISPR/Cas9	*BnaA9.WRKY47* contributed to adaptation to B deficiency by up-regulating BnaA3.NIP5;1 expression to facilitate efficient B uptake. Suggested BnaWRKY involvement in adaptation to low B	Feng et al., 2020
*B. napus*	Knockout mutations of all of *BnaFAD2* homologs	CRISPR/Cas9	Mutation type of *BnaFAD2* affected oleic levels varied, suggesting that fatty acid levels might be manipulated through precise editing of specific regions of a gene	Huang et al., 2020
*B. napus*	Generated diverse mutants of the male-sterility allele MS5a and MS5c	CRISPR/Cas9	Male fertility dependent on expression levels and protein sequences of MS5a and MS5c	Xin et al., 2020
*B. napus*	Created gain-of-function mutant of *BnaA6.RGA, bnaa6.rga-D*, and the loss-of-function quadruple mutant, *bnarga*	CRISPR/Cas9	Quadruple mutant bnarga significantly decreased the sensitivity of stomatal closure, suggesting that BnaRGA proteins play important roles in plant adaptation to water-deficit stress	Wu et al., 2020b
*B. napus*	Knockout members of the *BnSFAR4* and *BnSFAR5* gene families	CRISPR/Cas9	Significant increase of seed oil content	Karunarathna et al., 2020
*B. napus*	Induced calreticulin (*BnCRT1a*) mutants	CRISPR/Cas9	Loss of function of *CRT1a* results in activation of the ethylene signaling pathway. Observed reduced susceptibility to *Verticillium longisporum*	Pröbsting et al., 2020
*B. napus*	Knockout three functional paralogs of *BnITPK*	CRISPR/Cas9	Reduction of phytic acid and increase of free phosphorus in seed	Sashidhar et al., 2020
*B. napus*	Mutation of the *BnTT8* genes	CRISPR/Cas9	Yellow seed coat, altered oil content, and fatty acid composition in seeds	Zhai et al., 2020
**Targeted base editing**				
*B. napus*	C-to-T substitution at the P197 position of *BnALS 1*	Base editing	Tribenuron-methyl herbicide resistance	Wu et al., 2020a
*B. napus*	A-to-G substitutions in *BnALS* and *BnPDS*	Base editing	Single amino acid change in the FT protein or mis-splicing of the PDS3 RNA transcript resulted in late-flowering and albino phenotypes	Kang et al., 2018
*B. napus*	A-to-G substitutions in *ALS, RGA*, and *IAA7* genes	Base editing system	Base-edited plants of ALS conferred high herbicide resistance, while base-edited RGA or IAA7 plants exhibited decreased plant height	Cheng et al., 2020

*ZFP, zinc finger protein; TF, transcription factor; ZFN, zinc finger nuclease; CMS, cytoplasmic male sterility*.

#### Targeted Base Editing

Targeted base editing is one of the newest additions to GE. The technique is based on the CRISPR/Cas9 system and enables the direct and irremediable conversion of a selected target base without the induction and repair of a DSB (Komor et al., 2016). In the base-editing system, a cytosine or adenosine deaminase domain is fused to the N-terminus of a deactivated Cas9 (dCas9) of Cas9 nickase (nCas9). Although the Cas9 retains the ability to be guided by the gRNA, it instead mediates the direct conversion of cytidine (C) to uridine (U) resulting in a C-to-T or G-to-A substitution resulting in a single controlled point mutation or base correction rather than a random gene disruption. The precise mutations can lead to the introduction of stop codons, changes in amino acids, and regulatory site modification, thereby improving the resolution in the functional analysis of genes and proteins to a single nucleotide or amino acid (Komor et al., 2016). Kang et al. (2018) established an adenosine base editing (ABE) system in *B. napus*, demonstrating the efficiency of ABE in generating A-to-G substitutions at the target BnALS and BnPDS loci. The substitution resulted in a single amino acid change in the FT protein or mis-splicing of the PDS3 RNA transcript generating germline transmissible transgenic *Brassica* plants with late-flowering and albino phenotypes. The nCas9 cytosine base-editing system was employed by Wu et al. (2020a) to introduce a C-to-T conversion at the P197 position of the *BnALS 1* gene in *B. napus*. The P197S substitution conferred tribenuron-methyl resistance generating transgene-free homozygous mutants. Herbicide resistance and dwarfed plant architecture, both important traits in the commercial cultivation of oilseed rape, were reportedly introduced by Cheng et al. (2020) using the A3A-PBE base-editing system. The A3A-PBE base-editing system enabled the substitution of C to T with increased efficiency (>20%) and wider editing window.

Modifications brought upon by genome-editing technologies pose a significantly lower risk than those associated with transgenics. In general, only a few selected nucleotides are altered, rendering changes similar to those that occur in natural populations (Voytas and Gao, 2014). After the genomic-editing agents were segregated out, it is not possible to differentiate between a naturally occurring mutation and the gene edit. GE is therefore a valuable tool to establish rapid and precise changes to aid crop improvement (Zhang et al., 2018b).

## Genomic Selection in Crop Improvement

Plant breeding is founded on the principles of collection, induction, and rearrangement of genetic diversity followed by phenotypic-driven selection. Conventional breeding success was achieved through the exploitation of natural or mutation-induced variation followed by efficient selection of desirable traits largely based on phenotypic observation (Pérez-de-Castro et al., 2012). This approach has several limitations including long periods required (5–12 years) to develop a crop variety, high environmental noise, and being less effective in the improvement of complex and low heritable traits (Tuberosa, 2012).

Desirable traits often include characteristics such as increased yield, plant architecture, tolerance to environmental stresses, and resistance against pests and diseases. An interlinking network of multiple “minor” genes regulates the expression of these agronomically important features. Phenotypic expression is further shaped by non-genetic factors including genotype–environment interactions (Werner et al., 2018). It is not possible to accurately access these intricate and dynamic interdependencies based on phenotypic observation alone in conventional breeding. Crop development is therefore limited by extended periods of selections of up to 10 years, environmental noise, and low heredity of complex traits in conventional breeding approaches.

With the onset of the genomics era (Nepolean et al., 2018), marker-assisted breeding (MAS) was developed to address the limitations posed by conventional breeding (Collard and Mackill, 2008). In MAS, functional markers linked to QTL are used to detect important traits through linkage mapping or GWAS. As only statistically significant marker–trait associations are retained (Arruda et al., 2016), MAS is restricted to the detection of traits controlled by a limited number of QTL with large contributions to phenotypic variation. MAS has therefore limited value in the selection of traits under complex genetic control and is, as such, outperformed by traditional phenotypic selection (Bernardo, 2001, 2016; Zhao et al., 2014).

Fast-evolving genomic tools and vast amounts of available genomic sources are permitting the establishment of genotype–phenotype relationships, in particular for complex multi-genic traits (Pérez-de-Castro et al., 2012). Genome-wide selection or GS (Meuwissen et al., 2001), contrary to MAS, includes all marker information in the prediction model, reducing marker bias and allowing the potential to explain variance even with small-effect QTL. Predicted marker effects based on phenotype and high-density marker scores are then used to estimate the breeding value of untested genotypes (Zhao et al., 2015). This estimate, applied in the preselection of promising genotypes, can accelerate progress in crop breeding and reduce cost in comparison with conventional breeding (Wang X. et al., 2018).

Hybrid breeding has been widely used in the improvement of crop performance through the exploitation of heterosis (Liu et al., 2020). In heterosis, hybrid offspring created have the potential to outclass agronomic characteristics of the parents. The selection of suitable parental combinations is therefore of essence and can pose a major challenge in the development of hybrids. However, GS has shown the potential to predict hybrid performance (Zhao et al., 2015); resulting hybrid genotypes can be inferred from their inbred parents and potentially reduce genotyping cost and generation interval (Wang X. et al., 2018). GS methods for hybrid canola breeding was evaluated by Jan et al. (2016); genome-wide SNP profiles were used to evaluate the prediction of the best possible parental combination of pollinators crossed with the two tester lines in a testcross performance for a number of important traits in spring canola. Based on genome-wide SNP markers, it was determined that testcross performance prediction in canola breeding could be an effective and efficient method to preselect promising pollinators for combinations with available male-sterile maternal lines, thereby promoting the efficient allocation of breeding resources (Jan et al., 2016).

Würschum et al. (2014) investigated the potential of GS in rapeseed breeding reporting medium-to-high prediction accuracies for several morphological-, quality-, and yield-related traits. Despite lower accuracy in the prediction of some novel families, it was concluded that with increased marker availability, GS will provide a valuable genomic tool in knowledge-based rapeseed breeding. GS has further been applied in winter-type oilseed rape (Werner et al., 2017), spring-sown canola (Jan et al., 2016), and a biparental population based on a cross between a European winter cultivar and a Chinese semi-winter cultivar (Zou et al., 2016; Liu P. et al., 2017). Werner et al. (2018) investigated the value of marker selection approaches in Asian rapeseed and illustrated that high prediction accuracies for polygenic traits are achievable with low marker density, given that the representative markers were selected with regard to the genome-wide linkage disequilibrium (LD) structure in a population.

Increased phenotypic heritability has been shown to have a greater impact on whole-genome prediction accuracies, more so than training set population size and marker density (Zhang et al., 2017). Fikere et al. (2020) reported moderate-to-high genomic prediction accuracies using genomic best linear unbiased prediction (GBLUP) models upon evaluating genetic correlations and genomic prediction accuracies for several agronomic, disease, and seed quality traits in canola. The inclusion of genotype-by-environment interaction in the GBLUP model resulted in further, though slight, improvements in predictions. Koscielny et al. (2020) confirmed these findings, demonstrating higher accuracy in whole-genome predictions within the stress treatment than within the control treatment for the majority of traits evaluated. It is therefore important, even in the genomics era, to link selected phenotypic or demographic models with the underlying processes of genomic variation. As demonstrated in the CWR *Brassica cretica*, if variation is largely selectively neutral, it is not possible to assume that a diverse population will inescapably display the wide-ranging adaptive diversity required for further crop improvement (Kioukis et al., 2020).

## Future Prospects

Mechanisms of interaction for stress responses involve complex interactions and traits and are therefore more difficult to investigate than direct interaction (Werner et al., 2018). While genomic advances have exponentially increased during the past decades, high-throughput phenotyping has not caught up yet. To accelerate plant breeding and improve our understanding of genotype underlying expressed phenotype, dedicated high-throughput phenotyping approaches are required (Singh D. et al., 2019). It is therefore not surprising that high-throughput phenomics has increased in popularity, especially for the management and data collection of *Brassica* oilseed and vegetable crops. Improvements in sensor, drone, and remote sensing technology, as well as high throughput phenotyping techniques, are simplifying and enabling the quantification of complex phenotypic traits without the necessity of destructive sampling (Parmley et al., 2019). *Brassica* physiological studies, for example, plant height and biomass data (Moeckel et al., 2018), flower number (Wan et al., 2018), vegetation and flower fraction (Fang et al., 2016), and nitrogen nutrient studies (Graeff et al., 2008; Liu S. et al., 2018), have been generated using unmanned aerial vehicles. Assimilating large amounts of phenotypic data with the capabilities of machine learning will provide breeders with the analytical tools to optimize cultivar development in relation to target environment and accelerate the rate of genetic gain (Parmley et al., 2019).

Besides phenotypic characteristics, crop breeding requirements are also dictated by an assortment of additional major and minor variables such as environment, cultivation, and management practices and fluctuating consumer needs (Araus et al., 2018). Aligning breeding objectives to an increasing number of critical factors will require cross-disciplinary approaches driven by breeding teams, climate specialist, bioinfomaticians, and crop modelers (Beveridge et al., 2019; Stöckle and Kemanian, 2020). Crop modeling can assist breeders in comprehending the influence and interaction of variable factors in the selection of desirable varieties (Stöckle and Kemanian, 2020).

## Conclusion

Crop breeding has benefitted from the advancement of genomic tools and associated analysis pipelines. Available genomic resources and lower cost of high-throughput sequencing have contributed toward the increase in WGRS efforts. The vast amount of genomic information created and advances in genomic tools developed will significantly improve capturing the range of genetic diversity estimation and enhance the capturing and exploitation of diversity in *Brassica* germplasm profiles. The genetic libraries of CWRs should be further explored, as quality of available references and assembly methods has improved. The availability of GE tools has improved in precision and specificity; these systems are highly customizable and can be advantageously exploited to fast-track crop improvement. Although genomics is currently taking the center stage, a multidisciplinary plant breeding approach that includes phenotype = genotype × environment × management interaction backed by big data capabilities will ultimately ensure the selection of future-proof *Brassica* crops.

## Author Contributions

AS-E, NM, and JB conceptualized the manuscript. NM, AS-E, and AP wrote the manuscript, with additions and edits from JB and DE. The tables were prepared by NM and AS-E. AS-E illustrated the figure. All authors read and approved the final manuscript.

## Conflict of Interest

The authors declare that the research was conducted in the absence of any commercial or financial relationships that could be construed as a potential conflict of interest.

## References

[B1] Aghazadeh R. Zamani M. Motallebi M. Moradyar M. Moghadassi Jahromi Z. (2016). Co-transformation of canola by chimeric chitinase and *tlp* genes towards improving resistance to *Sclerotinia sclerotiorum*. World J. Microbiol. Biotechnol. 3:144. 10.1007/s11274-016-2104-6 27430511

[B2] Akama S. Shimizu-Inatsugi R. Shimizu K. K. Sese J. (2014). Genome-wide quantification of homeolog expression ratio revealed nonstochastic gene regulation in synthetic allopolyploid *Arabidopsis*. Nucleic Acids Res. 42:e46. 10.1093/nar/gkt1376 24423873 PMC3973336

[B3] Akter A. Takahashi S. Deng W. Shea D. J. Itabashi E. Shimizu M.. (2019). The histone modification H3 lysine 27 tri-methylation has conserved gene regulatory roles in the triplicated genome of *Brassica rapa* L. DNA Res. 26, 433–443. 10.1093/dnares/dsz021 31622476 PMC6796510

[B4] Allier A. Lehermeier C. Charcosset A. Moreau L. Teyssèdre S. (2019). Improving short- and long-term genetic gain by accounting for within-family variance in optimal cross-selection. Front. Genet. 10:1006. 10.3389/fgene.2019.01006 31737033 PMC6828944

[B5] Altpeter F. Springer N. M. Bartley L. E. Blechl A. E. Brutnell T. P. Citovsky V.. (2016). Advancing crop transformation in the era of genome editing. Plant Cell 28, 1510–1520. 10.1105/tpc.16.00196 27335450 PMC4981132

[B6] Álvarez-Venegas R. Zhang Y. Kraling K. Tulsieram L. (2011). Flowering without vernalization in winter canola (*Brassica napus*): use of Virus-Induced Gene Silencing (VIGS) to accelerate genetic gain. Nova Sci. 3, 29–50.

[B7] Amarasinghe S. L. Su S. Dong X. Zappia L. Ritchie M. E. Gouil Q. (2020). Opportunities and challenges in long-read sequencing data analysis. Genome Biol. 21:30. 10.1186/s13059-020-1935-5 32033565 PMC7006217

[B8] Amoah S. Kurup S. Rodriguez Lopez C. M. Welham S. J. Powers S. J. Hopkins C. J.. (2012). A hypomethylated population of *Brassica rapa* for forward and reverse epi-genetics. BMC Plant Biol. 12:193. 10.1186/1471-2229-12-193 23082790 PMC3507869

[B9] An H. Qi X. Gaynor M. L. Hao Y. Sarah C. Gebken S. C.. (2019). Transcriptome and organellar sequencing highlights the complex origin and diversification of allotetraploid *Brassica napus*. Nat. Commun. 10:2878. 10.1038/s41467-019-10757-1 31253789 PMC6599199

[B10] Araus J. L. Kefauver S. C. Zaman-Allah M. Olsen M. S. Cairns J. E. (2018). Translating high-throughput phenotyping into genetic gain. Trends Plant Sci. 23, 451–466. 10.1016/j.tplants.2018.02.001 29555431 PMC5931794

[B11] Arruda M. P. Lipka A. E. Brown P. J. Krill A. M. Thurber C. Brown-Guedira G.. (2016). Comparing genomic selection and marker-assisted selection for Fusarium head blight resistance in wheat (*Triticum aestivum* L.). Mol. Breed. 36:84. 10.1007/s11032-016-0508-5

[B12] Augustine R. Mukhopadhyay A. Bisht N. C. (2013). Targeted silencing of *BjMYB28* transcription factor gene directs development of low glucosinolate lines in oilseed *Brassica juncea*. Plant Biotechnol. J. 11, 855–866. 10.1111/pbi.12078 23721233

[B13] Backes G. (2013). TILLING and EcoTILLING, in Diagnostics in Plant Breeding, eds Lübberstedt T. Varshney R. K. (Dordrecht: Springer), 145–165.

[B14] Bancroft I. Morgan C. Fraser F. Higgins J. Wells R. Clissold L.. (2011). Dissecting the genome of the polyploid crop oilseed rape by transcriptome sequencing. Nat. Biotechnol. 29, 762–766. 10.1038/nbt.1926 21804563

[B15] Basso M. F. Arraes F. B. M. Grossi-de-Sa M. Moreira V. J. V. Alves-Ferreira M. Grossi-de-Sa M. F. (2020). Insights into genetic and molecular elements for transgenic crop development. Front. Plant Sci. 11:509. 10.3389/fpls.2020.00509 32499796 PMC7243915

[B16] Bayer P. E. Hurgobin B. Golicz A. A. Chan C.-K. K. Yuan Y. Lee H.. (2017). Assembly and comparison of two closely related *Brassica napus* genomes. Plant Biotechnol. J. 15, 1602–1610. 10.1111/pbi.12742 28403535 PMC5698052

[B17] Bebber D. P. Ramotowski M. A. T. Gurr S. J. (2013). Crop pests and pathogens move polewards in a warming world. Nat. Clim.Change 3, 985–988. 10.1038/nclimate1990

[B18] Becker H. C. Engqvist G. M. Karlsson B. (1995). Comparison of rapeseed cultivars and resynthesized lines based on allozyme and RFLP markers. Theor. Appl. Genet. 91, 62–67. 10.1007/BF00220859 24169668

[B19] Bekele D. Kassahun T. Fikre A. (2019). Applications of virus induced gene silencing (VIGS) in plant functional genomics studies. J. Plant Biochem. Physiol. 7:1. 10.4172/2329-9029.100022932481191

[B20] Belser C. Istace B. Denis E. Dubarry M. Baurens F.-C. Falentin C.. (2018). Chromosome-scale assemblies of plant genomes using nanopore long reads and optical maps. Nat. Plants 4, 879–887. 10.1038/s41477-018-0289-4 30390080

[B21] Bernardo R. (2001). What if we knew all the genes for a quantitative trait in hybrid crops? Crop Sci. 41, 1–4. 10.2135/cropsci2001.4111

[B22] Bernardo R. (2016). Bandwagons I, too, have known. Theor. Appl. Genet. 129, 2323–2332. 10.1007/s00122-016-2772-5 27681088

[B23] Beveridge L. Whitfield S. Challinor A. (2019). Crop modelling: towards locally relevant and climate-informed adaptation. Clim. Change 147, 475–489. 10.1007/s10584-018-2160-z

[B24] Beversdorf W. D. Hume D. J. Daonnelly-Vanderloo M. J. (1988). Agronomic performance of trianzine-resistant and susceptible reciprocal spring canola hybrids. Crop Sci. 28, 932–934. 10.2135/cropsci1988.0011183X002800060012x

[B25] Bizuayehu T. T. Kornel Labun K. Jefimov K. Valen E. (2020). Single molecule structure sequencing reveals RNA structural dependencies, breathing and ensembles. bioRxiv. 10.1101/2020.05.18.101402v1

[B26] Bodirsky B. L. Popp A. Weindl I. Dietrich J. P. Rolinski S. Scheiffele L.. (2012). N_2_O emissions from the global agricultural nitrogen cycle – current state and future scenarios. Biogeoscience 9, 4169–4197. 10.5194/bg-9-4169-2012

[B27] Braatz J. Harloff H. J. Mascher M. Stein N. Himmelbach A. Jung C. (2017). CRISPR-Cas9 targeted mutagenesis leads to simultaneous modification of different homoeologous gene copies in polyploid oilseed rape (*Brassica napus*). Plant Physiol. 174, 935–942. 10.1104/pp.17.00426 28584067 PMC5462057

[B28] Branca F. Argento S. Alessandro T. (2012). Assessing genetic reserves in Sicily (Italy): The *Brassica* wild relatives case study, in Agrobiodiversity Conservation: Securing the Diversity of Crop Wild Relatives and Landraces, eds Maxted N. Ehsan Dulloo M. Ford-Lloyd B. V. Frese L. Iriondo J. M. Pinheiro de Carvalho M. A. A. (Wallingford: Centre for Agriculture and Bioscience International), 52–58.

[B29] Briskine R. V. Paape T. Shimizu-Inatsugi R. Nishiyama T. Akama S. Sese J.. (2017). Genome assembly and annotation of *Arabidopsis halleri*, a model for heavy metal hyperaccumulation and evolutionary ecology. Mol. Ecol. Res. 17, 1025–1036. 10.1111/1755-0998.12604 27671113

[B30] Brookes G. Barfoot P. (2018). Farm income and production impacts of using GM crop technology 1996-2016. GM Crop. Food 9, 59–89. 10.1080/21645698.2018.1464866 29889608 PMC6277065

[B31] Brozynska M. Furtado A. Henry R. J. (2016). Genomics of crop wild relatives: expanding the gene pool for crop improvement. Plant Biotechnol. J. 14, 1070–1085. 10.1111/pbi.12454 26311018 PMC11389173

[B32] Budar F. Roux F. (2011). The role of organelle genomes in plant adaptation: time to get to work! Plant Signal. Behav. 6, 635–639. 10.4161/psb.6.5.14524 21499027 PMC3172827

[B33] Cai C. Wang X. Liu B. Wu J. Liang J. Cui Y.. (2017). *Brassica rapa* genome 2.0: a reference upgrade through sequence re-assembly and gene re-annotation. Mol. Plant. 10, 649–651. 10.1016/j.molp.2016.11.008 27890636

[B34] Cao J. Shelton A. M. Earle E. D. (2005). Development of transgenic collards (*Brassica oleracea* L., var. *acephala*) expressing a *cry*1Ac or *cry*1C Bt gene for control of the diamondback moth. Crop Prot. 24, 804–813. 10.1016/j.cropro.2004.12.014

[B35] Cappelli A. Cini E. (2020). Will the COVID-19 pandemic make us reconsider the relevance of short food supply chains and local productions? Trends Food Sci. Tech. 99, 566–567. 10.1016/j.tifs.2020.03.041 32288230 PMC7138154

[B36] Cermak T. Doyle E. L. Christian M. Wang L. Zhang Y. Schmidt C.. (2011). Efficient design and assembly of custom TALEN and other TAL effector-based constructs for DNA targeting. Nucleic Acids Res. 3:e82. 10.1093/nar/gkr218 21493687 PMC3130291

[B37] Chalhoub B. Denoeud F. Liu S. Parkin I. A. P. Tang H. Wang X.. (2014). Early allopolyploid evolution in the post-Neolithic *Brassica napus* oilseed genome. Science 34599, 950–953. 10.1126/science.1253435 25146293

[B38] Chang S. Yang T. Du T. Huang Y. Chen J. Yan J.. (2011). Mitochondrial genome sequencing helps show the evolutionary mechanism of mitochondrial genome formation in *Brassica*. BMC Genome 12:497. 10.1186/1471-2164-12-497 21988783 PMC3204307

[B39] Chen F. Yang Y. Li B. Liu Z. Khan F. Zhang T.. (2019). Functional analysis of M-Locus Protein Kinase revealed a novel regulatory mechanism of self-incompatibility in *Brassica napus* L. Int. J. Mol. Sci. 20:3303. 10.3390/ijms20133303 31284391 PMC6651594

[B40] Chen J. Guan R. Chang S. Du T. Zhang H. Xing H. (2011). Substoichiometrically different mitotypes coexist in mitochondrial genomes of *Brassica napus* L. PLoS ONE 6:e17662. 10.1371/journal.pone.0017662 21423700 PMC3053379

[B41] Chen X. Ge X. Wang J. Tan C. King G. J. Liu K. (2015). Genome-wide DNA methylation profiling by modified reduced representation bisulfite sequencing in *Brassica rapa* suggests that epigenetic modifications play a key role in polyploid genome evolution. Front. Plant Sci. 6:836. 10.3389/fpls.2015.00836 26500672 PMC4598586

[B42] Chen X. Tong C. Zhang X. Song A. Hu M. Dong W.. (2020). A high-quality *Brassica napus* genome reveals expansion of transposable elements, subgenome evolution and disease resistance. Plant Biotechnol. J. 10.1111/pbi.13493 33073445 PMC7955885

[B43] Chen Y. Zhou B. Li J. Tang H. Tang J. Yang Z. (2018). Formation and change of chloroplast-located plant metabolites in response to light conditions. Int. J. Mol. Sci. 19:654. 10.3390/ijms19030654 29495387 PMC5877515

[B44] Cheng F. Wu J. Cai C. Fu L. Liang J. Borm T.. (2016). Genome resequencing and comparative variome analysis in a *Brassica rapa* and *Brassica oleracea* collection. Sci. Data 3:160119. 10.1038/sdata.2016.119 27996963 PMC5170593

[B45] Cheng H. Hao M. Ding B. Mei D. Wang W. Wang H.. (2020). Base editing with high efficiency in allotetraploid oilseed rape by A3A-PBE base editing system. Plant Biotechnol. J. 19, 87–97. 10.1111/pbi.13444 32640102 PMC7769242

[B46] Chétrit P. Mathieu C. Muller J. P. Vedel F. (1984). Physical and gene mapping of cauliflower (*Brassica oleracea*) mitochondrial DNA. Curr. Genet. 8, 413–421. 10.1007/BF00433907 24177911

[B47] Clarke W. E. Higgins E. E. Plieske J. Wieseke R. Sidebottom C. Khedikar Y.. (2016). A high-density SNP genotyping array for *Brassica napus* and its ancestral diploid species based on optimised selection of single-locus markers in the allotetraploid genome. Theor. Appl. Genet. 129, 1887–1899. 10.1007/s00122-016-2746-7 27364915 PMC5025514

[B48] Clarke W. E. Parkin I. A. Gajardo H. A. Gerhardt D. J. Higgins E. Sidebottom C.. (2013). Genomic DNA enrichment using sequence capture microarrays: a novel approach to discover sequence nucleotide polymorphisms (SNP) in *Brassica napus* L. PLoS ONE 8:e081992. 10.1371/journal.pone.0081992 24312619 PMC3849492

[B49] Cokus S. J. Feng S. Zhang X. Chen Z. Merriman B. Haudenschild C. D.. (2008). Shotgun bisulphite sequencing of the *Arabidopsis* genome reveals DNA methylation patterning. Nature 452, 215–219. 10.1038/nature06745 18278030 PMC2377394

[B50] Collard B. C. Mackill D. J. (2008). Marker-assisted selection: an approach for precision plant breeding in the twenty-first century. Philos. Trans. R. Soc. B Biol. Sci. 363, 557–572. 10.1098/rstb.2007.2170 17715053 PMC2610170

[B51] Cong L. Ran F. A. Cox D. Lin S. Barretto R. Habib N.. (2013). Multiplex genome engineering using CRISPR/Cas systems. Science 339, 819–823. 10.1126/science.1231143 23287718 PMC3795411

[B52] Connelly M. MacIntosh S. (2018). Petition for Determination of Nonregulated Status for DHA Canola. Available online at: https://www.aphis.usda.gov/brs/aphisdocs/17_23601p.pdf

[B53] Cowling W. A. (2007). Genetic diversity in Australian canola and implications for crop breeding for changing future environments. Field Crop Res. 104, 103–111. 10.1016/j.fcr.2006.12.014

[B54] Cox D. B. T. Platt R. J. Zhang F. (2015). Therapeutic genome editing: prospects and challenges. Nat. Med. 21, 121–131. 10.1038/nm.3793 25654603 PMC4492683

[B55] Crutzen P. J. Ehhalt D. H. (1977). Effects of nitrogen fertilizers and combustion on the stratospheric ozone layer. Ambio 6, 112–117.

[B56] Cuthbert J. L. McVetty P. B. E. Freyssinet G. Freyssinet M. (2001). Comparison of the performance of bromoxynil resistant and susceptible near-isogenic populations of oilseed rape. Can. J. Plant Sci. 81, 367–372. 10.4141/P00-115

[B57] Dalton-Morgan J. Hayward A. Alamery S. Tollenaere R. Mason A. S. Campbell E.. (2014). A high-throughput SNP array in the amphidiploid species *Brassica napus* shows diversity in resistance genes. Funct. Integr. Genomics 14, 643–655. 10.1007/s10142-014-0391-2 25147024

[B58] Daniell H. (2007). Transgene containment by maternal inheritance: effective or elusive? Proc. Natl. Acad. Sci. U.S.A. 104, 6879–6880. 10.1073/pnas.0702219104 17440039 PMC1855423

[B59] Daniell H. Lin C.-S. Yu M. Chang W.-J. (2016). Chloroplast genomes: diversity, evolution, and applications in genetic engineering. Genome Biol. 17, 134–134. 10.1186/s13059-016-1004-2 27339192 PMC4918201

[B60] Darracq A. Varré J. S. Maréchal-Drouard L. Courseaux A. Castric V. Saumitou-Laprade P.. (2011). Structural and content diversity of mitochondrial genome in beet: a comparative genomic analysis. Genome Biol. Evol. 3, 723–736. 10.1093/gbe/evr042 21602571 PMC3163473

[B61] Das P. M. Ramachandran K. Van Wert J. Singal R. (2004). Chromatin immunoprecipitation assay. Biotech. 37, 961–969. 10.2144/04376RV01 15597545

[B62] Davis D. (2009). Declining fruit and vegetable nutrient composition: what is the evidence? Hortic. Sci. 44, 15–19. 10.21273/HORTSCI.44.1.15

[B63] Davis D. R. Epp M. D. Riordan H. D. (2005). Changes in USDA food composition data for 43 garden crops, 1950 to 1999. J. Am. Coll. Nutr. 23, 669–682. 10.1080/07315724.2004.10719409 15637215

[B64] De Block M. De Brouwer D. Tenning P. (1989). Transformation of *Brassica napus* and *Brassica oleracea* using *Agrobacterium tumefaciens* and the expression of the *bar* and *neo* genes in the transgenic plants. Plant Physiol. 91, 694–701. 10.1104/pp.91.2.694 16667089 PMC1062058

[B65] de Paulo Farias D. dos Santos Gomes M. G. (2020). COVID-19 outbreak: what should be done to avoid food shortages? Trends Food Sci. Technol. 102, 291–292. 10.1016/j.tifs.2020.06.007 32834501 PMC7293531

[B66] Dempewolf H. Baute G. Anderson J. Kilian B. Smith C. Guarino L. (2017). Past and future use of wild relatives in crop breeding. Crop Sci. 57, 1070–1082. 10.2135/cropsci2016.10.0885

[B67] Dirks R. van Dun K. de Snoo C. B. van den Berg M. Lelivelt C. L. Voermans W.. (2009). Reverse breeding: a novel breeding approach based on engineered meiosis. Plant Biotechnol. J. 7, 837–845. 10.1111/j.1467-7652.2009.00450.x 19811618 PMC2784905

[B68] Dolatabadian A. Bayer P. E. Tirnaz S. Hurgobin B. Edwards D. Batley J. (2020). Characterization of disease resistance genes in the *Brassica napus* pangenome reveals significant structural variation. Plant Biotechnol. J. 18, 969–982. 10.1111/pbi.13262 31553100 PMC7061875

[B69] Dolinoy D. C. Weinhouse C. Jones T. R. Rozek L. S. Jirtle R. L. (2010). Variable histone modifications at the A_*vy*_ metastable epiallele. Epigenetics. 5, 637–644. 10.4161/epi.5.7.12892 20671424 PMC3052847

[B70] Dreccer M. F. Schapendonk A. H. C. M. Slafer G. A. Rabbinge R. (2000). Comparative response of wheat and oilseed rape to nitrogen supply: absorption and utilisation efficiency of radiation and nitrogen during the reproductive stages determining yield. Plant Soil 220, 189–205. 10.1023/A:1004757124939

[B71] Dusenge M. E. Duarte A. G. Way D. A. (2019). Plant carbon metabolism and climate change: elevated CO_2_ and temperature impacts on photosynthesis, photorespiration and respiration. New Phytol. 221, 32–49. 10.1111/nph.15283 29983005

[B72] Dwivedi S. L. Scheben A. Edwards D. Spillane C. Ortiz R. (2017). Assessing and exploiting functional diversity in germplasm pools to enhance abiotic stress adaptation and yield in cereals and food legumes. Front. Plant Sci. 8:1461. 10.3389/fpls.2017.01461 28900432 PMC5581882

[B73] Elhamamsy A. R. (2016). DNA methylation dynamics in plants and mammals: overview of regulation and dysregulation. Cell Biochem. Funct. 34, 289–298. 10.1002/cbf.3183 27003927

[B74] Espinoza C. Schlechter R. Herrera D. Torres E. Serrano A. Medina C.. (2013). Cisgenesis and intragenesis: new tools for improving crops. Biol. Res. 46, 323–331. 10.4067/S0716-97602013000400003 24510134

[B75] Fang S. Tang W. Peng Y. Gong Y. Dai C. Chai R.. (2016). Remote estimation of vegetation fraction and flower fraction in oilseed rape with unmanned aerial vehicle data. Remote Sens. 8:416. 10.3390/rs8050416

[B76] Feng Y. Cui R. Wang S. He M. Hua Y. Shi L.. (2020). Transcription factor BnaA9.WRKY47 contributes to the adaptation of *Brassica napus* to low boron stress by up-regulating the boric acid channel gene *BnaA3.NIP5;1*. Plant Biotechnol. J. 18, 1241–1254. 10.1111/pbi.13288 31705705 PMC7152615

[B77] Ferreira de Carvalho J. Lucas J. Deniot G. Falentin C. Filangi O. Gilet M.. (2019). Cytonuclear interactions remain stable during allopolyploid evolution despite repeated whole-genome duplications in *Brassica*. Plant J. 98, 434–447. 10.1111/tpj.14228 30604905

[B78] Ficklin D. L. Novick K. A. (2017). Historic and projected changes in vapor pressure deficit suggest a continental-scale drying of the United States atmosphere. J. Geophys. Res. Atmos. 122, 2061–2079. 10.1002/2016jd025855

[B79] Fikere M. Barbulescu D. M. Malmberg M. M. Maharjan P. Salisbury P. A. Kant S.. (2020). Genomic prediction and genetic correlation of agronomic, blackleg disease, and seed quality traits in canola (*Brassica napus* L.). Plants 9:719. 10.3390/plants9060719 32517116 PMC7356366

[B80] Florez-Sarasa I. Fernie A. R. Gupta K. J. (2020). Does the alternative respiratory pathway offer protection against the adverse effects resulting from climate change? J. Exp. Bot. 71, 465–469. 10.1093/jxb/erz428 31559421 PMC6946008

[B81] Francisco M. Tortosa M. Martínez-Ballesta M. d,.C Velasco P. García-Viguera C.. (2017). Nutritional and phytochemical value of *Brassica* crops from the agri-food perspective. Ann. Appl. Biol. 170, 273–285. 10.1111/aab.12318

[B82] Frewer L. J. van der Lans I. Fischer A. R. H. Reinders M. J. Menozzi D. Zhang X.. (2013). Public perceptions of agri-food applications of genetic modification – A systematic review and meta-analysis. Trends Food Sci. Technol. 30, 142–152. 10.1016/j.tifs.2013.01.003

[B83] Fu Y-B. Gugel R. K. (2010). Genetic diversity of Canadian elite summer rape (Brassica napus L.) cultivars from the pre- to post-canola quality era. Can. J. Plant Sci. 90, 23–33. 10.4141/CJPS09073

[B84] Fuchs J. Demidov D. Houben A. Schubert I. (2006). Chromosomal histone modification patterns – from conservation to diversity. Trends Plant Sci. 11, 199–208. 10.1016/j.tplants.2006.02.008 16546438

[B85] Gabur I. Chawla H. S. Lopisso D. T. von Tiedemann A. Snowdon R. J. Obermeier C. (2020). Gene presence-absence variation associates with quantitative *Verticillium longisporum* disease resistance in *Brassica napus*. Sci. Rep. 10:4131. 10.1038/s41598-020-61228-3 32139810 PMC7057980

[B86] Gabur I. Chawla H. S. Snowdon R. J. Parkin I. A. P. (2018). Connecting genome structural variation with complex traits in crop plants. Theor. Appl. Genet. 132, 733–750. 10.1007/s00122-018-3233-0 30448864

[B87] Gaebelein R. Schiessl S. V. Samans B. Batley J. Mason A. S. (2019). Inherited allelic variants and novel karyotype changes influence fertility and genome stability in *Brassica* allohexaploids. New Phytol. 223, 965–978. 10.1111/nph.15804 30887525

[B88] Gaj T. Gersbach C. A. Barbas C. F. (2013). ZFN, TALEN, and CRISPR/Cas-based methods for genome engineering. Trends Biotechnol. 31, 397–405. 10.1016/j.tibtech.2013.04.004 23664777 PMC3694601

[B89] Gajardo H. A. Wittkop B. Soto-Cerda B. Higgins E. E. Parkin I. A. P. Snowdon R. J.. (2015). Association mapping of seed quality traits in *Brassica napus* L. using GWAS and candidate QTL approaches. Mol. Breed. 35:143. 10.1007/s11032-015-0340-3

[B90] Gallusci P. Dai Z. Génard M. Gauffretau A. Leblanc-Fournier N. Richard-Molard C.. (2017). Epigenetics for plant improvement: current knowledge and modeling avenues. Trends Plant Sci. 22, 610–623. 10.1016/j.tplants.2017.04.009 28587758

[B91] Garg M. Sharma N. Sharma S. Kapoor P. Kumar A. Chunduri V.. (2018). Biofortified crops generated by breeding, agronomy, and transgenic approaches are improving lives of millions of people around the world. Front. Nutr. 5:12. 10.3389/fnut.2018.00012 29492405 PMC5817065

[B92] Gazave E. Tassone E. E. Ilut D. C. Wingerson M. Datema E.. (2016). Population genomic analysis reveals differential evolutionary histories and patterns of diversity across subgenomes and subpopulations of *Brassica napus* L. Front. Plant Sci. 7:525. 10.3389/fpls.2016.00525 27148342 PMC4838616

[B93] Geng X. X. Chen S. Astarini I. A. Yan G. J. Tian E. Meng J. L.. (2013). Doubled haploids of novel trigenomic *Brassica* derived from various interspecific crosses. Plant Cell Tissue Organ Cult. 113, 501–511. 10.1007/s11240-013-0292-4

[B94] Gilchrist E. J. Sidebottom C. H. D. Koh C. S. MacInnes T. Sharpe A. G. Haughn G. W. (2013). A mutant *Brassica napus* (canola) population for the identification of new genetic diversity via TILLING and next generation sequencing. plos ONE 8:e84303. 10.1371/journal.pone.0084303 24376800 PMC3869819

[B95] Gocal G. F. W. Schöpke C. Beetham P. R. (2015). Oligo-Mediated Targeted Gene Editing, in Advances in New Technology for Targeted Modification of Plant Genomes, eds Zhang F. Puchta H. Thomson J. (New York, NY: Springer), 73–89.

[B96] Golicz A. A. Bayer P. E. Barker G. C. Edger P. P. Kim H. Martinez P. A.. (2016). The pangenome of an agronomically important crop plant *Brassica oleracea*. Nat. Commun. 7:13390. 10.1038/ncomms13390 27834372 PMC5114598

[B97] Graeff S. Pfenning J. Claupein W. Liebig H.-P. (2008). Evaluation of image analysis to determine the N-fertilizer demand of broccoli plants (*Brassica oleracea* convar. botrytis var. italica). Adv. Opt. Tech. 2008, 1–8. 10.1155/2008/359760

[B98] Grewe F. Edger P. P. Keren I. Sultan L. Pires J. C. Ostersetzer-Biran O.. (2014). Comparative analysis of 11 Brassicales mitochondrial genomes and the mitochondrial transcriptome of *Brassica oleracea*. Mitochondrion 19, 135–143. 10.1016/j.mito.2014.05.008 24907441

[B99] Grison R. Grezes-Besset B. Schneider M. Lucante N. Olsen L. Leguay J. J.. (1996). Field tolerance to fungal pathogens of *Brassica napus* constitutively expressing a chimeric chitinase gene. Nat. Biotechnol. 14, 643–646. 10.1038/nbt0596-643 9630959

[B100] Guo X. Hu Q. Hao G. Wang X. Zhang D. Ma T.. (2018). The genomes of two *Eutrema* species provide insight into plant adaptation to high altitudes. DNA Res. 25, 307–315. 10.1093/dnares/dsy003 29394339 PMC6014361

[B101] Gupta M. Atri C. Agarwal N. Banga S. S. (2016). Development and molecular-genetic characterization of a stable *Brassica* allohexaploid. Theor. Appl. Genet. 129, 2085–2100. 10.1007/s00122-016-2759-2 27480156

[B102] Gupta M. DeKelver R. C. Palta A. Clifford C. Gopalan S. Miller J. C.. (2012). Transcriptional activation of *Brassica napus* β-ketoacyl-ACP synthase II with an engineered zinc finger protein transcription factor. Plant Biotechnol. J. 10, 783–791. 10.1111/j.1467-7652.2012.00695.x 22520333

[B103] Han F. Zhang X. Liu X. Su H. Kong C. Fang Z.. (2017). Comparative analysis of genome wide DNA methylation profiles for the genic male sterile cabbage line 01-20S and its maintainer line. Genes 8:159. 10.3390/genes8060159 28621722 PMC5485523

[B104] Handa H. (2003). The complete nucleotide sequence and RNA editing content of the mitochondrial genome of rapeseed (*Brassica napus* L.): Comparative analysis of the mitochondrial genomes of rapeseed and *Arabidopsis thaliana*. Nucleic Acids Res. 31, 5907–5916. 10.1093/nar/gkg795 14530439 PMC219474

[B105] Harlan J. R. (1992). Crops and Man. 2nd Edn. Madison, WI: American Society of Agronomy and Crop Science Society of America. 10.2135/1992

[B106] Harloff H.-J. Lemcke S. Mittasch J. Frolov A. Wu J. G. Dreyer F.. (2012). A mutation screening platform for rapeseed (*Brassica napus* L.) and the detection of sinapine biosynthesis mutants. Theor. Appl. Genet. 124, 957–969. 10.1007/s00122-011-1760-z 22198204

[B107] Harper A. L. Trick M. Higgins J. Fraser F. Clissold L. Wells R.. (2012). Associative transcriptomics of traits in the polyploid crop species *Brassica napus*. Nat. Biotechnol. 30, 798–802. 10.1038/nbt.2302 22820317

[B108] Hatono S. Nishimura K. Murakami Y. Tsujimura M. Yamagishi H. (2017). Complete mitochondrial genome sequences of *Brassica rapa* (Chinese cabbage and mizuna), and intraspecific differentiation of cytoplasm in B. rapa and Brassica juncea. Breed. Sci. 67, 357–362. 10.1270/jsbbs.17023 29085245 PMC5654463

[B109] Hauben M. Haesendonckx B. Standaert E. Van Der Kelen K. Azmi A. Akpo H.. (2009). Energy use efficiency is characterized by an epigenetic component that can be directed through artificial selection to increase yield. Proc. Natl. Acad. Sci. U.S.A. 106, 20109–20114. 10.1073/pnas.0908755106 19897729 PMC2774259

[B110] He Y. Wu D. Wei D. Fu Y. Cui Y. Dong H.. (2017). GWAS, QTL mapping and gene expression analyses in *Brassica napus* reveal genetic control of branching morphogenesis. Sci. Rep. 7:15971. 10.1038/s41598-017-15976-4 29162897 PMC5698412

[B111] He Z. Cheng F. Li Y. Wang X. Parkin I.A. Chalhoub B.. (2015). Construction of Brassica A and C genome-based ordered pan-transcriptomes for use in rapeseed genomic research. Data Brief 4, 357–362. 10.1016/j.dib.2015.06.016 26217816 PMC4510581

[B112] Heffner E. L. Sorrells M. E. Jannink J.-L. (2009). Genomic selection for crop improvement. Crop Sci. 49, 1–12. 10.2135/cropsci2008.08.0512

[B113] Heng S. Wang L. Yang X. Huang H. Chen G. Cui M.. (2020). Genetic and comparative transcriptome analysis revealed degs involved in the purple leaf formation in *Brassica juncea*. Front. Genet. 11:322. 10.3389/fgene.2020.00322 32391051 PMC7193680

[B114] Herrera R. J. Garcia-Bertrand R. (eds.). (2018). The agricultural revolutions, in Ancestral DNA, Human Origins, and Migrations (London: Academic Press), 475–509.

[B115] Hickey L. T. Hafeez A. N. Robinson H. Jackson S. A. Leal-Bertioli S. Tester M.. (2019). Breeding crops to feed 10 billion. Nat. Biotechnol. 37, 744–754. 10.1038/s41587-019-0152-9 31209375

[B116] Higgins E. E. Clarke W. E. Howell E. C. Armstrong S. J. Parkin I. A. P. (2018). Detecting *de novo* homoeologous recombination events in cultivated *Brassica napus* using a genome-wide SNP array. G3 8, 2673–2683. 10.1534/g3.118.200118 29907649 PMC6071606

[B117] Himelblau E. Gilchrist E. J. Buono K. Bizzell C. Mentzer L. Vogelzang R.. (2009). Forward and reverse genetics of rapid-cycling *Brassica oleracea*. Theor. Appl. Genet. 118, 953–961. 10.1007/s00122-008-0952-7 19132334

[B118] Holme I. B. K. Wendt T. Holm P. B. (2013). Intragenesis and cisgenesis as alternatives to transgenic crop development. Plant Biotechnol. J. 11, 395–407. 10.1111/pbi.12055 23421562

[B119] Hong H. Datla N. Reed D. W. Covello P. S. MacKenzie S. L. Qiu X. (2002). High-level production of γ-linolenic acid in *Brassica juncea* using a Δ6 desaturase from *Pythium irregulare*. Plant Physiol. 129, 354–362. 10.1104/pp.001495 12011365 PMC155898

[B120] Houlton B. Z. Almaraz M. Aneja V. Austin A. T. Bai E. Cassman K. G.. (2019). A world of cobenefits: solving the global nitrogen challenge. Earths Future 7, 865–872. 10.1029/2019EF001222 PMC6733275 31501769

[B121] Hu H. Scheben A. Edwards D. (2018). Advances in integrating genomics and bioinformatics in the plant breeding pipeline. Agriculture 8:75. 10.3390/agriculture8060075

[B122] Hu T. T. Pattyn P. Bakker E. G. Cao J. Cheng J.-F. Clark R. M.. (2011). The *Arabidopsis lyrata* genome sequence and the basis of rapid genome size change. Nat. Genet. 43, 476–481. 10.1038/ng.807 21478890 PMC3083492

[B123] Hu Z.-Y. Hua W. Huang S.-M. Wang H.-Z. (2011). Complete chloroplast genome sequence of rapeseed (*Brassica napus* L.) and its evolutionary implications. Genet. Resour. Crop Evol. 58, 875–887. 10.1007/s10722-010-9626-9

[B124] Huang F. Liu T. Hou X. (2018). Isolation and functional characterization of a floral repressor, *BcMAF1*, from Pak-choi (*Brassica rapa* ssp. chinensis). Front. Plant Sci. 9:290. 10.3389/fpls.2018.00290 29559991 PMC5845726

[B125] Huang H. Cui T. Zhang L. Yang Q. Yang Y. Xie K.. (2020). Modifications of fatty acid profile through targeted mutation at *BnaFAD2* gene with CRISPR/Cas9-mediated gene editing in *Brassica napus*. Theor. Appl. Genet. 133, 2401–2411. 10.1007/s00122-020-03607-y 32448919

[B126] Hurgobin B. Edwards D. (2017). SNP discovery using a pangenome: has the single reference approach become obsolete? Biology 6:21. 10.3390/biology6010021 28287462 PMC5372014

[B127] Hurgobin B. Golicz A. A. Bayer P. E. Chan C.-K. K. Tirnaz S. Dolatabadian A.. (2018). Homoeologous exchange is a major cause of gene presence/absence variation in the amphidiploid *Brassica napus*. Plant Biotechnol. J. 16, 1265–1274. 10.1111/pbi.12867 29205771 PMC5999312

[B128] Hyseni L. Bromley H. Kypridemos C. O'Flaherty M. Lloyd-Williams F. Guzman-Castillo M.. (2017). Systematic review of dietary trans-fat reduction interventions. Bull. World Health Organ. 95, 821G−830G. 10.2471/BLT.16.189795 29200523 PMC5710076

[B129] Jan H. U. Abbadi A. Lücke S. Nichols R. A. Snowdon R. J. (2016). Genomic prediction of testcross performance in canola (*Brassica napus*). PLoS ONE 11:e0147769. 10.1371/journal.pone.0147769 26824924 PMC4732662

[B130] Jansing J. Schiermeyer A. Schillberg S. Fischer R. Bortesi L. (2019). Genome editing in agriculture: Technical and practical considerations. Int. J. Mol. Sci. 20:2888. 10.3390/ijms20122888 31200517 PMC6627516

[B131] Jiang L. Li D. Jin L. Ruan Y. Shen W.-H. Liu C. (2018). Histone lysine methyltransferases BnaSDG 8.A and BnaSDG 8.C are involved in the floral transition in *Brassica napus*. Plant J. 95, 672–685. 10.1111/tpj.13978 29797624

[B132] Jin M. Liu H. He C. Fu J. Xiao Y. Wanget Y.. (2016). Maize pan-transcriptome provides novel insights into genome complexity and quantitative trait variation. Sci. Rep. 6:18936. 10.1038/srep18936 26729541 PMC4733048

[B133] Jin S. Daniell H. (2015). The engineered chloroplast genome just got smarter. Trends Plant Sci. 20, 622–640. 10.1016/j.tplants.2015.07.004 26440432 PMC4606472

[B134] Jinek M. Chylinski K. Fonfara I. Hauer M. Doudna J. A. Charpentier E. (2012). A programmable dual-RNA-guided DNA endonuclease in adaptive bacterial immunity. Science 337, 816–821. 10.1126/science.1225829 22745249 PMC6286148

[B135] Jump A. S. Peñuelas J. (2005), Running to stand still: adaptation the response of plants to rapid climate change. Ecol. Lett. 8, 1010–1020. 10.1111/j.1461-0248.2005.00796.x 34517682

[B136] Jung H.-J. Jung H.-J. Ahmed N. U. Park J.-I. Kang K.-K. Hur Y.. (2012). Development of self-compatible *B. rapa* by RNAi-mediated S locus gene silencing. PLoS ONE 7:e49497. 10.1371/journal.pone.0049497 23145180 PMC3493532

[B137] Kamthan A. Chaudhuri A. Kamthan M. Datta A. (2016). Genetically modified (GM) crops: milestones and new advances in crop improvement. Theor. Appl. Genet. 129, 1639–1655. 10.1007/s00122-016-2747-6 27381849

[B138] Kanazawa A. Inaba J-i. Kasai M. Shimura H. Masuta C. (2011). RNA-mediated epigenetic modifications of an endogenous gene targeted by a viral vector. Plant Signal. Behav. 6, 1090–1093. 10.4161/psb.6.8.16046 21772121 PMC3260699

[B139] Kang B. Yun J. Kim S. Shin Y. Ryu J. Choi M.. (2018). Precision genome engineering through adenine base editing in plants. Nat. Plants 4, 427–431. 10.1038/s41477-018-0178-x 29867128

[B140] Karunarathna N. L. Wang H. Harloff H. J. Jiang L. Jung C. (2020). Elevating seed oil content in a polyploid crop by induced mutations in *SEED FATTY ACID REDUCER* genes. Plant Biotechnol. J. 18:11. 10.1111/pbi.13381 32216029 PMC7589255

[B141] Kasianov A. S. Klepikova A. V. Kulakovskiy I. V. Gerasimov E. S. Fedotova A. V. Besedina E. G.. (2017). High-quality genome assembly of *Capsella bursa-pastoris* reveals asymmetry of regulatory elements at early stages of polyploid genome evolution. Plant J. 91, 278–291. 10.1111/tpj.13563 28387959

[B142] Kawanabe T. Osabe K. Itabashi E. Okazaki K. Dennis E. S. Fujimoto R. (2016). Development of primer sets that can verify the enrichment of histone modifications, and their application to examining vernalization-mediated chromatin changes in *Brassica rapa* L. Genes Genet. Syst. 91:1. 10.1266/ggs.15-00058 27074983

[B143] Kazama T. Okuno M. Watari Y. Yanase S. Koizuka C. Tsuruta Y.. (2019). Curing cytoplasmic male sterility via TALEN-mediated mitochondrial genome editing. Nat. Plants 5, 722–730. 10.1038/s41477-019-0459-z 31285556

[B144] Khan A. W. Garg V. Roorkiwal M. Golicz A. A. Edwards D. Varshney R. K. (2020). Super-pangenome by integrating the wild side of a species for accelerated crop improvement. Trends Plant Sci. 25, 148–158. 10.1016/j.tplants.2019.10.012 31787539 PMC6988109

[B145] Khan S. U. Yangmiao J. Liu S. Zhang K. Khan M. H. U. Zhai Y.. (2019). Genome-wide association studies in the genetic dissection of ovule number, seed number, and seed weight in *Brassica napus* L. Ind. Crop Prod. 142:111877. 10.1016/j.indcrop.2019.111877

[B146] Khush G. S. Lee S. Cho J.-I. Jeon J.-S. (2012). Biofortification of crops for reducing malnutrition. Plant Biotechnol. Rep. 6, 195–202. 10.1007/s11816-012-0216-5

[B147] Kim J. A. Jung H. E. Hong J. K. Hermand V. McClung C. R. Lee Y. H.. (2016). Reduction of GIGANTEA expression in transgenic *Brassica rapa* enhances salt tolerance. Plant Cell Rep. 35, 1943–1954. 10.1007/s00299-016-2008-9 27295265

[B148] Kim Y. G. Cha J. Chandrasegaran S. (1996). Hybrid restriction enzymes: zinc finger fusions to FokI cleavage domain. Proc. Natl Acad. Sci. U.S.A. 93, 1156–1160. 10.1073/pnas.93.3.1156 8577732 PMC40048

[B149] Kioukis A. Michalopoulou V. A. Briers L. Pirintsos S. Studholme D. J. Pavlidis P.. (2020). Intraspecific diversification of the crop wild relative *Brassica cretica* Lam. using demographic model selection. BMC Genomics 21:48. 10.1186/s12864-019-6439-x 31937246 PMC6961386

[B150] Kirchner T.W. Niehaus M. Rössig K.L. Lauterbach T. Herde M. Küster H.. (2018). Molecular background of Pi deficiency-induced root hair growth in *Brassica carinata*?a fasciclin-like arabinogalactan protein is involved. Front. Plant Sci. 9:1372. 10.3389/fpls.2018.01372 30283481 PMC6157447

[B151] Kitashiba H. Li F. Hirakawa H. Kawanabe T. Zou Z. Hasegawa Y.. (2014). Draft sequences of the radish (*Raphanus sativus* L.) genome. DNA Res. 21, 481–490. 10.1093/dnares/dsu014 24848699 PMC4195494

[B152] Kode V. Mudd E. A. Iamtham S. Day A. (2005). The tobacco plastid *accD* gene is essential and is required for leaf development. Plant J. 44, 237–244. 10.1111/j.1365-313X.2005.02533.x 16212603

[B153] Komor A. C. Kim Y. B. Packer M. S. Zuris J. A. Liu D. R. (2016). Programmable editing of a target base in genomic DNA without double-stranded DNA cleavage. Nature 533, 420–424. 10.1038/nature17946 27096365 PMC4873371

[B154] Koscielny C. B. Gardner S. W. Technow F. Duncan R. W. (2020). Linkage mapping and whole-genome predictions in canola (*Brassica napus*) subjected to differing temperature treatments. Crop Pasture Sci. 71, 229–238. 10.1071/CP19387

[B155] Kumar M. S. Mawlong I. Rani R. (2020). Biofortification of *Brassicas* for quality improvement, in Brassica Improvement: Molecular, Genetics and Genomic Perspectives, eds Wani S. H. Thakur A. K. Jeshima Khan Y. (Cham: Springer International Publishing), 127–145.

[B156] Lawrenson T. Shorinola O. Stacey N. Li C. Østergaard L. Patron N.. (2015). Induction of targeted, heritable mutations in barley and *Brassica oleracea* using RNA-guided Cas9 nuclease. Genome Biol. 16:258. 10.1186/s13059-015-0826-7 26616834 PMC4663725

[B157] Lee H. Chawla H. S. Obermeier C. Dreyer F. Abbadi A. Snowdon R. (2020). Chromosome-scale assembly of winter oilseed rape *Brassica napus*. Front. Plant Sci. 11:496. 10.3389/fpls.2020.00496 32411167 PMC7202327

[B158] Leff B. Ramankutty N. Foley J. A. (2004). Geographic distribution of major crops across the world. Glob. Biogeochem. Cycles 18:1. 10.1029/2003gb002108

[B159] Li C. Hao M. Wang W. Wang H. Chen F. Chu W.. (2018). An efficient CRISPR/Cas9 platform for rapidly generating simultaneous mutagenesis of multiple gene homoeologs in allotetraploid oilseed rape. Front. Plant Sci. 9:442. 10.3389/fpls.2018.00442 29731757 PMC5920024

[B160] Li H. Cheng X. Zhang L. Hu J. Zhang F. Chen B.. (2018). An integration of genome-wide association study and gene co-expression network analysis identifies candidate genes of stem lodging-related traits in *Brassica napus*. Front. Plant Sci. 9:796. 10.3389/fpls.2018.00796 29946333 PMC6006280

[B161] Li J. Rao L. Meng Q. Ghani M. A. Chen L. (2015). Production of Brassica tri-genomic vegetable germplasm by hybridisation between tuber mustard (*Brassica juncea*) and red cabbage (*B. oleracea*). Euphytica 204, 323–333. 10.1007/s10681-014-1336-5

[B162] Li L. Liu Y. Chen B. Xu K. Zhang F. Li H.. (2016). A genome-wide association study reveals new loci for resistance to clubroot disease in *Brassica napus*. Front. Plant Sci. 7:1483. 10.3389/fpls.2016.01483 27746804 PMC5044777

[B163] Li P. Zhang S. Li F. Zhang S. Zhang H. Wang X.. (2017). A phylogenetic analysis of chloroplast genomes elucidates the relationships of the six economically important *Brassica* species comprising the Triangle of U. Front. Plant Sci. 8:111. 10.3389/fpls.2017.00111 28210266 PMC5288352

[B164] Li T. Lui B. Spalding M. H. Weeks D. P. Yang B. (2012). High-efficiency TALEN-based gene editing produces disease-resistant rice. Nat. Biotechnol. 30, 390–392. 10.1038/nbt.2199 22565958

[B165] Li Y. Liu G-F. Ma L-M. Liu T-K. Zhang C-W. Xiao D.. (2020). A chromosome-level reference genome of non-heading Chinese cabbage [*Brassica campestris* (syn. *Brassica rapa*) ssp. chinensis]. Hortic. Res. 7:212. 10.1038/s41438-020-00449-z 33372175 PMC7769993

[B166] Li Y. Tollefsbol T. O. (2011). Combined chromatin immunoprecipitation and bisulfite methylation sequencing analysis. Methods Mol. Biol. 791, 239–251. 10.1007/978-1-61779-316-5_18 21913084 PMC3233221

[B167] Lima M. S. Woods L. C. Cartwright M. W. Smith D. R. (2016). The (in)complete organelle genome: exploring the use and nonuse of available technologies for characterizing mitochondrial and plastid chromosomes. Mol. Ecol. Res. 16, 1279–1286. 10.1111/1755-0998.12585 27482846

[B168] Limera C. Sabbadini S. Sweet J. B. Mezzetti B. (2017). New biotechnological tools for the genetic improvement of major woody fruit species. Front. Plant Sci. 8:1418. 10.3389/fpls.2017.01418 28861099 PMC5559511

[B169] Lin J. Musunuru K. (2016). Genome engineering tools for building cellular models of disease. FEBS J. 283, 3222–3231. 10.1111/febs.13763 27218233 PMC5881911

[B170] Lin K. Zhang N. Severing E. I. Nijveen H. Cheng F. Visser R. G. F.. (2014). Beyond genomic variation - comparison and functional annotation of three *Brassica rapa* genomes: a turnip, a rapid cycling and a Chinese cabbage. BMC Genomics 15:250. 10.1186/1471-2164-15-250 24684742 PMC4230417

[B171] Lister R. O'Malley R. C. Tonti-Filippini J. Gregory B. D. Berry C. C. Millar A. H.. (2008). Highly integrated single-base resolution maps of the epigenome in *Arabidopsis*. Cell 133, 523–536. 10.1016/j.cell.2008.03.029 18423832 PMC2723732

[B172] Liu C. W. Lin C. C. Yiu J. C. Chen J. J. Tseng M. J. (2008). Expression of a *Bacillus thuringiensis* toxin (*cry1Ab*) gene in cabbage (*Brassica oleracea* L. *var. capitata* L.) chloroplasts confers high insecticidal efficacy against *Plutella xylostella*. Theor. Appl. Genet. 117, 75–88. 10.1007/s00122-008-0754-y 18415072

[B173] Liu G. Xia Y. Liu T. Dai S. Hou X. (2018). The DNA methylome and association of differentially methylated regions with differential gene expression during heat stress in *Brassica rapa*. Int. J. Mol. Sci. 19:1414. 10.3390/ijms19051414 29747401 PMC5983725

[B174] Liu J. Li M. Zhang Q. Wei X. Huang X. (2020). Exploring the molecular basis of heterosis for plant breeding. J. Integr. Plant Biol. 62, 287–298. 10.1111/jipb.12804 30916464

[B175] Liu P. Zhao Y. Liu G. Wang M. Hu D. Hu J.. (2017). Hybrid performance of an immortalized F_2_ rapeseed population is driven by additive, dominance, and epistatic effects. Front. Plant Sci. 8:815. 10.3389/fpls.2017.00815 28572809 PMC5435766

[B176] Liu S. Li L. Gao W. Zhang Y. Liu Y. Wang S.. (2018). Diagnosis of nitrogen status in winter oilseed rape (*Brassica napus* L.) using *in-situ* hyperspectral data and unmanned aerial vehicle (UAV) multispectral images. Comput. Electron. Agric. 151, 185–195. 10.1016/j.compag.2018.05.026

[B177] Liu S. Liu Y. Yang X. Tong C. Edwards D. Parkin I. A. P.. (2014). The *Brassica oleracea* genome reveals the asymmetrical evolution of polyploid genomes. Nat. Commun. 5:3930. 10.1038/ncomms4930 24852848 PMC4279128

[B178] Liu T. Li Y. Duan W. Huang F. Hou X. (2017). Cold acclimation alters DNA methylation patterns and confers tolerance to heat and increases growth rate in *Brassica rapa*. J. Exp. Bot. 68, 1213–1224. 10.1093/jxb/erw496 28158841 PMC5441862

[B179] Lowe R. Shirley N. Bleackley M. Dolan S. Shafee T. (2017). Transcriptomics technologies. PLoS Comput. Biol. 13:e1005457. 10.1371/journal.pcbi.1005457 28545146 PMC5436640

[B180] Lu G. Harper A. L. Trick M. Morgan C. Fraser F. O'Neill C.. (2014). Associative transcriptomics study dissects the genetic architecture of seed glucosinolate content in *Brassica napus*. DNA Res. 21, 613–625. 10.1093/dnares/dsu024 25030463 PMC4263295

[B181] Lu K. Wei L. Li X. Wang Y. Wu J. Liu M.. (2019). Whole-genome resequencing reveals *Brassica napus* origin and genetic loci involved in its improvement. Nat. Commun. 10:1154. 10.1038/s41467-019-09134-9 30858362 PMC6411957

[B182] Lu R. Martin-Hernandez A. M. Peart J. R. Malcuit I. Baulcombe D. C. (2003). Virus-induced gene silencing in plants. Methods 30, 296–303. 10.1016/S1046-2023(03)00037-9 12828943

[B183] Lu S. Van Eck J. Zhou X. Lopez A. B. O'Halloran D. M. Cosman K. M.. (2006). The cauliflower *Or* gene encodes a DnaJ cysteine-rich domain-containing protein that mediates high levels of β-carotene accumulation. Plant Cell 18, 3594–3605. 10.1105/tpc.106.046417 17172359 PMC1785402

[B184] Lu Y. Yao J. (2018). Chloroplasts at the crossroad of photosynthesis, pathogen infection and plant defense. Int. J. Mol. Sci. 19:3900. 10.3390/ijms19123900 30563149 PMC6321325

[B185] Lusser M. Davies H. V. (2013). Comparative regulatory approaches for groups of new plant breeding techniques. New Biotechnol. 30, 437–446. 10.1016/j.nbt.2013.02.004 23474021

[B186] Lusser M. Parisi C. Plan D. Rodríguez-Cerezo E. (2012). Deployment of new biotechnologies in plant breeding. Nat. Biotechnol. 30, 231–239. 10.1038/nbt.2142 22398616 PMC7097357

[B187] Lv H. Wang Y. Han F. Ji J. Fang Z. Zhuang M.. (2020). A high-quality reference genome for cabbage obtained with SMRT reveals novel genomic features and evolutionary characteristics. Sci. Rep. 10:12394. 10.1038/s41598-020-69389-x 32709963 PMC7381634

[B188] Ma C. Zhu C. Zheng M. Liu M. Zhang D. Liu B.. (2019). CRISPR/Cas9-mediated multiple gene editing in *Brassica oleracea* var. *capitata* using the endogenous tRNA-processing system. Hortic. Res. 6:20. 10.1038/s41438-018-0107-1 30729010 PMC6355899

[B189] Makaroff C. A. Palmer J. D. (1987). Extensive mitochondrial specific transcription of the *Brassica campestris* mitochondrial genome. Nucleic Acids Res. 15, 5141–5156. 10.1093/nar/15.13.5141 3601669 PMC305952

[B190] Malek M. Rahman L. Das M. Hassan L. Rafii M. (2013). Development of hexaploid 'Brassica' (AABBCC) from hybrids (ABC) of '*Brassica carinata'* (BBCC) x *B. rapa* (AA). Aust. J. Crop Sci. 7, 1375–1382.

[B191] Malmberg M. M. Shi F. Spangenberg G. C. Daetwyler H. D. Cogan N. O. I. (2018). Diversity and genome analysis of Australian and global oilseed *Brassica napus* L. germplasm using transcriptomics and whole genome re-sequencing. Front. Plant Sci. 9:508. 10.3389/fpls.2018.00508 29725344 PMC5917405

[B192] Marconi G. Pace R. Traini A. Raggi L. Lutts S. Chiusano M.. (2013). Use of MSAP markers to analyse the effects of salt stress on DNA methylation in rapeseed (*Brassica napus* var. oleifera). PLoS ONE. 8:e75597. 10.1371/journal.pone.0075597 24086583 PMC3781078

[B193] Marri P. R. Ye L. Jia Y. Jiang K. Rounsley S. D. (2018). Advances in sequencing and resequencing in crop plants, in Plant Genetics and Molecular Biology, eds Varshney R. K. Pandey M. K. Chitikineni A. (Cham: Springer International Publishing), 11–35. 10.1007/10_2017_46 29516115

[B194] Mascher M. Schreiber M. Scholz U. Graner A. Reif J. C. Stein N. (2019). Genebank genomics bridges the gap between the conservation of crop diversity and plant breeding. Nat. Genet. 51, 1076–1081. 10.1038/s41588-019-0443-6 31253974

[B195] Mayer A. (1997). Historical changes in the mineral content of fruits and vegetables. Br. Food J. 99, 207–211. 10.1108/00070709710181540

[B196] McCallum C. M. Comai L. Greene E. A. Henikoff S. (2000). Targeting Induced Local Lesions IN Genomes (TILLING) for plant functional genomics. Plant Physiol. 123, 439–442. 10.1104/pp.123.2.439 10859174 PMC1539256

[B197] Mei J. Shao C. Yang R. Feng Y. Gao Y. Ding Y.. (2020). Introgression and pyramiding of genetic loci from wild *Brassica oleracea* into *B. napus* for improving Sclerotinia resistance of rapeseed. Theor. Appl. Genet. 133, 1313–1319. 10.1007/s00122-020-03552-w 32008057

[B198] Meuwissen T. H. Hayes B. J. Goddard M. E. (2001). Prediction of total genetic value using genome-wide dense marker maps. Genetics 157, 1819–1829. 11290733 10.1093/genetics/157.4.1819 PMC1461589

[B199] Michael T. P. Jupe F. Bemm F. Motley S. T. Sandoval J. P. Lanz C.. (2018). High contiguity *Arabidopsis thaliana* genome assembly with a single nanopore flow cell. Nat. Commun. 9:541. 10.1038/s41467-018-03016-2 29416032 PMC5803254

[B200] Ming R. Man Wai C. (2015). Assembling allopolyploid genomes: no longer formidable. Genome Biol. 16:27. 10.1186/s13059-015-0585-5 25723730 PMC4312463

[B201] Moeckel T. Dayananda S. Nidamanuri R. R. Nautiyal S. Hanumaiah N. Buerkert A.. (2018). Estimation of vegetable crop parameter by multi-temporal UAV-borne images. Remote Sens. 10:805. 10.3390/rs10050805

[B202] Moghe G. D. Hufnagel D. E. Tang H. Xiao Y. Dworkin I. Town C. D.. (2014). Consequences of whole-genome triplication as revealed by comparative genomic analyses of the wild radish *Raphanus raphanistrum* and three other Brassicaceae species. Plant Cell 26, 1925–1937. 10.1105/tpc.114.124297 24876251 PMC4079359

[B203] Moloney M. M. Walker J. M. Sharma K. K. (1989). High efficiency transformation of *Brassica napus* using *Agrobacterium* vectors. Plant Cell Rep. 8, 238–242. 10.1007/BF00778542 24233146

[B204] Murray G. M. Brennan J. P. (2012). The Current and Potential Costs From Diseases of Oilseed Crops in Australia. Grains Research and Development Corporation. Available online at: https://grdc.com.au/__data/assets/pdf_file/0021/82641/grdcreportdiseasecostoilseedspdf.pdf.pdf

[B205] Mwathi M. W. Gupta M. Quezada-Martinez D. Pradhan A. Batley J. Mason A. S.. (2020). Fertile allohexaploid *Brassica* hybrids obtained from crosses between *B. oleracea* and *B. juncea* via ovule rescue and colchicine treatment of cuttings. Plant Cell Tissue Organ Cult. 140, 301–313. 10.1007/s11240-019-01728-x

[B206] Napier J. A. Olsen R. E. Tocher D. R. (2019). Update on GM canola crops as novel sources of omega-3 fish oils. Plant Biotechnol. J. 17, 703–705. 10.1111/pbi.13045 30485634 PMC6419714

[B207] Navarro-León E. Ruiz J. M. Graham N. Blasco B. (2018). Physiological profile of CAX1a TILLING mutants of *Brassica rapa* exposed to different calcium doses. Plant Sci. 272, 164–172. 10.1016/j.plantsci.2018.04.019 29807588

[B208] Nepolean T. Kaul J. Mukri G. Mittal S. (2018). Genomics-enabled next-generation breeding approaches for developing system-specific drought tolerant hybrids in maize. Front. Plant Sci. 9:361. 10.3389/fpls.2018.00361 29696027 PMC5905169

[B209] Okuzaki A. Ogawa T. Koizuka C. Kaneko K. Inaba M. Imamura J.. (2018). CRISPR/Cas9-mediated genome editing of the fatty acid desaturase 2 gene in *Brassica napus*. Plant Physiol. Biochem. 131, 63–69. 10.1016/j.plaphy.2018.04.025 29753601

[B210] Palmer J. D. Herbon L. A. (1986). Tricircular mitochondrial genomes of *Brassica* and *Raphanus*: Reversal of repeat configurations by inversion. Nucleic Acids Res. 14, 9755–9764. 10.1093/nar/14.24.9755 3027662 PMC341333

[B211] Palmer J. D. Herbon L. A. (1988). Plant mitochondrial DNA evolved rapidly in structure, but slowly in sequence. J. Mol. Evol. 28, 87–97. 10.1007/BF02143500 3148746

[B212] Palmer J. D. Shields C. R. (1984). Tripartite structure of the *Brassica campestris* mitochondrial genome. Nature 307, 437–440. 10.1038/307437a0

[B213] Paritosh K. Yadava S. K. Singh P. Bhayana L. Mukhopadhyay A. Gupta V.. (2020). A chromosome-scale assembly of allotetraploid *Brassica juncea* (AABB) elucidates comparative architecture of the A and B genomes. Plant Biotechnol. J. 10.1111/pbi.13492 33073461 PMC7955877

[B214] Parkin I. A. P. Koh C. Tang H. Robinson S. J. Kagale S. Clarke W. E.. (2014). Transcriptome and methylome profiling reveals relics of genome dominance in the mesopolyploid *Brassica oleracea*. Genome Biol. 15:R77. 10.1186/gb-2014-15-6-r77 24916971 PMC4097860

[B215] Parmley K. A. Higgins R. H. Ganapathysubramanian B. Sakar S. Singh A. K. (2019). Machine learning approach for prescriptive plant breeding. Sci. Rep. 9:17132. 10.1038/s41598-019-53451-4 31748577 PMC6868245

[B216] Paszkowski J. Whitham S. A. (2001). Gene silencing and DNA methylation processes. Curr. Opin. Plant Biol. 4, 123–129. 10.1016/S1369-5266(00)00147-3 11228434

[B217] Payá-Milans M. Poza-Viejo L. Martín-Uriz P. S. Lara-Astiaso D. Wilkinson M. D. Crevillén P. (2019). Genome-wide analysis of the H3K27me3 epigenome and transcriptome in *Brassica rapa*. GigaScience 8:12. 10.1093/gigascience/giz147 31800038 PMC6892454

[B218] Pe'ery T. Mathews M. B. Baulcombe D. (2003). RNA interference. Methods 30, 287–288. 10.1016/S1046-2023(03)00035-5

[B219] Pérez-de-Castro A. M. Vilanova S. Cañizares J. Pascual L. Blanca J. M. Díez M. J.. (2012). Application of genomic tools in plant breeding. Curr. Genomics 13, 179–195. 10.2174/138920212800543084 23115520 PMC3382273

[B220] Perrella G. Kaiserli E. (2016). Light behind the curtain: Photoregulation of nuclear architecture and chromatin dynamics in plants. New Phytol. 212, 908–919. 10.1111/nph.14269 27813089 PMC5111779

[B221] Perumal S. Koh C. S. Jin L. Buchwaldt M. Higgins E. E. Zheng C.. (2020). A high-contiguity *Brassica nigra* genome localizes active centromeres and defines the ancestral *Brassica* genome. Nat. Plants 6, 929–941. 10.1038/s41477-020-0735-y 32782408 PMC7419231

[B222] Prabhudas S. K. Raju B. Kannan Thodi S. Parani M. Natarajan P. (2016). The complete chloroplast genome sequence of Indian mustard (*Brassica juncea* L.). Mitochondrial DNA Part A DNA Mapp. Seq. Anal. 27, 4622–4623. 10.3109/19401736.2015.1101586 26708222

[B223] Pradhan A. Plummer J. A. Nelson M. N. Cowling W. A. Yan G. (2010). Successful induction of trigenomic hexaploid *Brassica* from a triploid hybrid of *B. napus* L. and *B. nigra* (L.) Koch. Euphytica 176:87–98. 10.1007/s10681-010-0218-8

[B224] Pröbsting M. Schenke D. Hossain R. Häder C. Thurau T. Wighardt L.. (2020). Loss of function of CRT1a (calreticulin) reduces plant susceptibility to *Verticillium longisporum* in both *Arabidopsis thaliana* and oilseed rape (*Brassica napus*). Plant Biotechnol. J. 18, 2328–2344. 10.1111/pbi.13394 32358986 PMC7589372

[B225] Rahman H. (2013). Review: Breeding spring canola (*Brassica napus* L.) by the use of exotic germplasm. Can. J. Plant Sci. 93, 363–373. 10.4141/cjps2012-074

[B226] Rakyan V. K. Blewitt M. E. Druker R. Preis J. I. Whitelaw E. (2002). Metastable epialleles in mammals. Trends Genet. 18, 348–351. 10.1016/S0168-9525(02)02709-9 12127774

[B227] Raman H. Uppal R. K. Raman R. (2019). Genetic solutions to improve resilience of canola to climate change, in Genomic Designing of Climate-Smart Oilseed Crops, ed Kole C. (Cham: Springer International Publishing), 75–131.

[B228] Ramegowda V. Mysore K. S. Senthil-Kumar M. (2014). Virus-induced gene silencing is a versatile tool for unraveling the functional relevance of multiple abiotic-stress-responsive genes in crop plants. Front. Plant Sci. 5:323. 10.3389/fpls.2014.00323 25071806 PMC4085877

[B229] Ran Y. Liang Z. Gao C. (2017). Current and future editing reagent delivery systems for plant genome editing. Sci. China Life Sci. 60, 490–505. 10.1007/s11427-017-9022-1 28527114

[B230] Rando O. J. Ahmad K. (2007). Rules and regulation in the primary structure of chromatin. Curr. Opin. Cell Biol. 19, 250–256. 10.1016/j.ceb.2007.04.006 17466507

[B231] Rao V. R. Hodgkin T. (2002). Genetic diversity and conservation and utilization of plant genetic resources. Plant Cell Tissue Organ Cult. 68, 1–19. 10.1023/A:1013359015812

[B232] Rashid M. He G. Guanxiao Y. Ziaf K. (2011). Relevance of TILLING in plant genomics. Aust. J. Crop Sci. 5, 411–420.

[B233] Ray D. K. West P. C. Clark M. Gerber J. S. Prishchepov A. V. Chatterjee S. (2019). Climate change has likely already affected global food production. PLoS ONE 14:e0217148. 10.1371/journal.pone.0217148 31150427 PMC6544233

[B234] Razzaq A. Saleem F. Kanwal M. Mustafa G. Yousaf S. Imran Arshad H. M.. (2019). Modern trends in plant genome editing: an inclusive review of the CRISPR/Cas9 toolbox. Int. J. Mol. Sci. 20:4045. 10.3390/ijms20164045 31430902 PMC6720679

[B235] Richards E. J. (2006). Inherited epigenetic variation — revisiting soft inheritance. Nat. Rev. Genet. 7, 395–401. 10.1038/nrg1834 16534512

[B236] Rousseau-Gueutin M. Belser C. Da Silva C. Richard G. Istace B. Cruaud C.. (2020). Long-reads assembly of the *Brassica napus* reference genome, Darmor-bzh. GigaScience 9:12. 10.1093/gigascience/giaa137 33319912 PMC7736779

[B237] Ruiter R. van den Brande I. Stals E. Delauré S. Cornelissen M. D'Halluin K. (2003). Spontaneous mutation frequency in plants obscures the effect of chimeraplasty. Plant Mol. Biol. 53, 675–689. 10.1023/B:PLAN.0000019111.96107.01 15010606

[B238] Saini P. Saini P. Kaur J. J. Francies R. M. Gani M. Rajendra A. A.. (2020). Molecular approaches for harvesting natural diversity for crop improvement, in Rediscovery of Genetic and Genomic Resources for Future Food Security, eds Salgotra R. K. Zargar S. M. (Singapore: Springer), 67–169.

[B239] Sang S. Cheng H. Mei D. Fu L. Wang H. Liu J.. (2020). Complete organelle genomes of *Sinapis arvensis* and their evolutionary implications. Crop J. 8, 505–514. 10.1016/j.cj.2019.12.001

[B240] Sasaki T. Fujimoto R. Kishitani S. Nishio T. (2011). Analysis of target sequences of DDM1s in *Brassica rapa* by MSAP. Plant Cell Rep. 30, 81–88. 10.1007/s00299-010-0946-1 21072521

[B241] Sashidhar N. Harloff H.-J. Jung C. (2019). Identification of phytic acid mutants in oilseed rape (*Brassica napus*) by large-scale screening of mutant populations through amplicon sequencing. New Phytol. 225:2022–2034. 10.1111/nph.16281 31651044

[B242] Sashidhar N. Harloff H. J. Potgieter L. Jung C. (2020). Gene editing of three *BnITPK* genes in tetraploid oilseed rape leads to significant reduction of phytic acid in seeds. Plant Biotechnol. J. 18, 2241–2250. 10.1111/pbi.13380 32191373 PMC7589381

[B243] Sauer N. J. Narváez-Vásquez J. Mozoruk J. Miller R. B. Warburg Z. J. Woodward M. J.. (2016). Oligonucleotide-mediated genome editing provides precision and function to engineered nucleases and antibiotics in plants. Plant Physiol. 170, 1917–1928. 10.1104/pp.15.01696 26864017 PMC4825113

[B244] Saxena K. B. Hingane A. J. (2015). Male sterility systems in major field crops and their potential role in crop improvement, in Plant Biology and Biotechnology, eds Bahadur B. Venkat Rajam M. Sahijram L. Krishnamurthy K. (New Delhi: Springer), 639–656.

[B245] Scheben A. Berpaalen B. Lawley C. T. Chan C. K. K. Bayer P. E. Batley J.. (2019). CropSNPdb: a database of SNP array data for Brassica crops and hexaploid bread wheat. Plant J. 98, 142–152. 10.1111/tpj.14194 30548723

[B246] Scheben A. Wolter F. Batley J. Puchta H. Edwards D. (2017). Towards CRISPR/Cas crops bringing together genomics and genome editing. New Phytol. 216, 682–698. 10.1111/nph.14702 28762506

[B247] Scheben A. Yuan Y. Edwards D. (2016). Advances in genomics for adopting crops to climate change. Curr. Plant Biol. 6, 2–10. 10.1016/j.cpb.2016.09.001

[B248] Scholze H. Boch J. (2010). TAL effector-DNA specificity. Virulence 1:428–432. 10.4161/viru.1.5.12863 21178484

[B249] Schreiber M. Stein N. Mascher M. (2018). Genomic approaches for studying crop evolution. Genome Biol. 19:140. 10.1186/s13059-018-1528-8 30241487 PMC6151037

[B250] Schuler T. H. Denholm I. Clark S. J. Stewart C. N. Poppy G. M. (2004). Effects of Bt plants on the development and survival of the parasitoid *Cotesia plutellae* (Hymenoptera: Braconidae) in susceptible and Bt-resistant larvae of the diamondback moth, *Plutella xylostella* (Lepidoptera: Plutellidae). J. Insect Physiol. 50, 435–443. 10.1016/j.jinsphys.2004.03.001 15121457

[B251] Senthil-Kumar M. Mysore K. S. (2011). New dimensions for VIGS in plant functional genomics. Trends Plant Sci. 16, 656–665. 10.1016/j.tplants.2011.08.006 21937256

[B252] Seol Y.-J. Kim K. Kang S.-H. Perumal S. Lee J. Kim C.-K. (2017). The complete chloroplast genome of two *Brassica* species, *Brassica nigra* and *B. oleracea*. Mitochondrial DNA Part A DNA Mapp. Seq. Appl. 28, 167–168. 10.3109/19401736.2015.1115493 26709541

[B253] Seymour D. K. Becker C. (2017). The causes and consequences of DNA methylome variation in plants. Curr. Opin. Plant Biol. 36, 56–63. 10.1016/j.pbi.2017.01.005 28226269

[B254] Sharafi Y. Majidi M. M. Goli S. A. H. Rashidi F. (2015). Oil content and fatty acids composition in *Brassica* species. Int. J. Food Prop. 18, 2145–2154. 10.1080/10942912.2014.968284

[B255] Shen X. Xu L. Liu Y. Dong H. Zhou D. Zhang Y.. (2019). Comparative transcriptome analysis and ChIP-sequencing reveals stage-specific gene expression and regulation profiles associated with pollen wall formation in *Brassica rapa*. BMC Genomics 20:264. 10.1186/s12864-019-5637-x 30943898 PMC6446297

[B256] Shiba H. Kakizaki T. Iwano M. Tarutani Y. Watanabe M. Isogai A.. (2006). Dominance relationships between self-incompatibility alleles controlled by DNA methylation. Nat. Genet. 38, 297–299. 10.1038/ng1734 16444272

[B257] Shirasawa K. Hirakawa H. Fukino N. Kitashiba H. Isobe S. (2020). Genome sequence and analysis of a Japanese radish (*Raphanus sativus*) cultivar named ‘Sakurajima Daikon’ possessing giant root. DNA Res. 27:2. 10.1093/dnares/dsaa010 32426809 PMC7334891

[B258] Siche R. (2020). What is the impact of COVID-19 disease on agriculture? Sci. Agrop. 11, 3–6. 10.17268/sci.agropecu.2020.01.00

[B259] Singh D. Wang X. Kumar U. Gao L. Noor M. Imtiaz M.. (2019). High-throughput phenotyping enabled genetic dissection of crop lodging in wheat. Front. Plant Sci. 10:394. 10.3389/fpls.2019.00394 31019521 PMC6459080

[B260] Singh N. Wu S. Raupp W. J. Sehgal S. Arora S. Tiwari V.. (2019). Efficient curation of genebanks using next generation sequencing reveals substantial duplication of germplasm accessions. Sci. Rep. 9:650. 10.1038/s41598-018-37269-0 30679756 PMC6346010

[B261] Slatkin M. (2008). Linkage disequilibrium? understanding the evolutionary past and mapping the medical future. Nat. Rev. Genet. 9, 477–485. 10.1038/nrg2361 18427557 PMC5124487

[B262] Slotte T. Hazzouri K. M. Ågren J. A. Koenig D. Maumus F. Guo Y.-L.. (2013). The *Capsella rubella* genome and the genomic consequences of rapid mating system evolution. Nat. Genet. 45, 831–835. 10.1038/ng.2669 23749190

[B263] Snowdon R. J. Friedrich T. Friedt W. Köhler W. (2002). Identifying the chromosomes of the A- and C-genome diploid *Brassica* species *B. rapa (syn. campestris)* and *B. oleracea* in their amphidiploid *B. napus*. Theor. Appl. Genet. 104, 533–538. 10.1007/s00122-001-0787-y 12582655

[B264] Solís M. T. El-Tantawy A. A. Cano V. Risueño M. C. Testillano P. S. (2015). 5-azacytidine promotes microspore embryogenesis initiation by decreasing global DNA methylation, but prevents subsequent embryo development in rapeseed and barley. Front. Plant Sci. 6:472. 10.3389/fpls.2015.00472 26161085 PMC4479788

[B265] Song J.-M. Guan Z. Hu J. Guo C. Yang Z. Wang S.. (2020). Eight high-quality genomes reveal pan-genome architecture and ecotype differentiation of *Brassica napus*. Nat. Plants 6, 34–45. 10.1038/s41477-019-0577-7 31932676 PMC6965005

[B266] Sottosanto J. Andre C. Arias D. I. Bhatti M. Breazeale S. Fu H.. (2018). Petition for the Determination of Non-regulatory Status for EPA+*DHA Canola Event LBFLFK*. Available online at: https://www.aphis.usda.gov/brs/aphisdocs/17_32101p.pdf

[B267] Stephenson P. Baker D. Girin T. Perez A. Amoah S. King G. J.. (2010). A rich TILLING resource for studying gene function in *Brassica rapa*. BMC Plant Biol. 10:62. 10.1186/1471-2229-10-62 20380715 PMC2923536

[B268] Stöckle C. O. Kemanian A. R. (2020). Can crop models identify critical gaps in genetics, environment, and management interactions? Front. Plant Sci. 11:737. 10.3389/fpls.2020.00737 32595666 PMC7303354

[B269] Sun D. Wang C. Zhang X. Zhang W. Jiang H. Yao X.. (2019). Draft genome sequence of cauliflower (*Brassica oleracea* L. *var. botrytis*) provides new insights into the C genome in *Brassica* species. Hortic. Res. 6:82. 10.1038/s41438-019-0164-0 31645943 PMC6804732

[B270] Sun F. Fan G. Hu Q. Zhou Y. Guan M. Tong C.. (2017). The high-quality genome of *Brassica napus* cultivar ‘ZS11’ reveals the introgression history in semi-winter morphotype. Plant J. 92, 452–468. 10.1111/tpj.13669 28849613

[B271] Sun Q. Lin L. Liu D. Wu D. Fang Y. Wu J.. (2018). CRISPR/Cas9-mediated multiplex genome editing of the *BnWRKY11* and *BnWRKY70* genes in *Brassica napus* L. Int. J. Mol. Sci. 19:2716. 10.3390/ijms19092716 30208656 PMC6163266

[B272] Sun Z. Li N. Huang G. Xu J. Pan Y. Wang Z.. (2013). Site-specific gene targeting using transcription activator-like effector (TALE)-based nuclease in *Brassica oleracea*. J. Integr. Plant Biol. 55, 1092–1103. 10.1111/jipb.12091 23870552

[B273] Supple M. A. Shapiro B. (2018). Conservation of biodiversity in the genomics era. Genome Biol. 19:131. 10.1186/s13059-018-1520-3 30205843 PMC6131752

[B274] Sweetman C. Waterman C. D. Rainbird B. M. Smith P. Jenkins C. D. Day D. A.. (2019). *AtNDB2* is the main external NADH dehydrogenase in mitochondria and is important for tolerance to environmental stress. Plant Physiol. 181, 774–788. 10.1104/pp.19.00877 31409698 PMC6776847

[B275] Takahashi S. Fukushima N. Osabe K. Itabashi E. Shimizu M. Miyaji N.. (2018a). Identification of DNA methylated regions by using methylated DNA immunoprecipitation sequencing in *Brassica rapa*. Crop Pasture Sci. 69, 107–120. 10.1071/CP17394

[B276] Takahashi S. Osabe K. Fukushima N. Takuno S. Miyaji N. Shimizu M.. (2018b). Genome-wide characterization of DNA methylation, small RNA expression, and histone H3 lysine nine di-methylation in *Brassica rapa* L. DNA Res. 25, 511–520. 10.1093/dnares/dsy021 29982343 PMC6191303

[B277] Tan C. Liu H. Ren J. Ye X. Feng H. Liu Z. (2019). Single-molecule real-time sequencing facilitates the analysis of transcripts and splice isoforms of anthers in Chinese cabbage (*Brassica rapa* L. ssp. *pekinensis*). BMC Plant Biol. 1:517. 10.1186/s12870-019-2133-z 31771515 PMC6880451

[B278] Tanaka Y. Tsuda M. Yasumoto K. Terachi T. Yamagishi H. (2014). The complete mitochondrial genome sequence of *Brassica oleracea* and analysis of coexisting mitotypes. Curr. Genet. 60, 277–284. 10.1007/s00294-014-0433-2 24916859

[B279] Tang T. Yu X. Yang H. Gao Q. Ji H. Wang Y.. (2018). Development and validation of an effective CRISPR/Cas9 vector for efficiently isolating positive transformants and transgene-free mutants in a wide range of plant species. Front. Plant Sci. 9:1533. 10.3389/fpls.2018.01533 30405669 PMC6206294

[B280] Tanksley S. D. McCouch S. R. (1997). Seed banks and molecular maps: unlocking genetic potential from the wild. Science 277, 1063–1066. 10.1126/science.277.5329.1063 9262467

[B281] Tariq M. Paszkowski J. (2004). DNA and histone methylation in plants. Trends Genet. 20, 244–251. 10.1016/j.tig.2004.04.005 15145577

[B282] Tennessen J. A. Govindarajulu R. Ashman T.-L. Liston A. (2014). Evolutionary origins and dynamics of octoploid strawberry subgenomes revealed by dense targeted capture linkage maps. Genome Biol. Evol. 6, 3295–3313. 10.1093/gbe/evu261 25477420 PMC4986458

[B283] Tettelin H. Masignani V. Cieslewicz M. J. Donati C. Medini D. Ward N. L.. (2005). Genome analysis of multiple pathogenic isolates of *Streptococcus agalactiae*: implications for the microbial “pan-genome”. Proc. Natl. Acad. Sci. U.S.A. 102, 13950–13955. 10.1073/pnas.0506758102 16172379 PMC1216834

[B284] The Arabidopsis Genome Initiative (2000). Analysis of the genome sequence of the flowering plant *Arabidopsis thaliana*. Nature 408, 796–815. 10.1038/35048692 11130711

[B285] Tian E. Jiang Y. Chen L. Liu F. Menget J. (2010). Synthesis of a *Brassica* trigenomic allohexaploid (*B. carinata* × *B. rapa) de novo* and its stability in subsequent generations. Theor. Appl. Genet. 121, 1431–1440. 10.1007/s00122-010-1399-1 20607208

[B286] Tirnaz S. Batley J. (2019a). DNA methylation: toward crop disease resistance improvement. Trends Plant Sci. 24, 1137–1150. 10.1016/j.tplants.2019.08.007 31604599

[B287] Tirnaz S. Batley J. (2019b). Epigenetics: potentials and challenges in crop breeding. Mol Plant 12, 1309–1311. 10.1016/j.molp.2019.09.006 31541738

[B288] Tirnaz S. Merce C. Bayer P. E. Severn-Ellis A. A. Edwards D. Batley J. (2020). Effect of *Leptosphaeria maculans* infection on promoter DNA methylation of defence genes in *Brassica napus*. Agronomy 10:1072. 10.3390/agronomy10081072

[B289] Townsend A. R. Howarth R. W. Bazzaz F. A. Booth M. S. Cleveland C. C. Collinge S. K.. (2003). Human health effects of a changing global nitrogen cycle. Front. Ecol. Environ. 1, 240–246. 10.1890/1540-9295(2003)001[0240:HHEOAC]2.0.CO;2

[B290] Tuberosa R. (2012). Phenotyping for drought tolerance of crops in the genomics era. Front. Physiol. 3:347. 10.3389/fphys.2012.00347 23049510 PMC3446691

[B291] Nagaharu U. Nagaharu N. Nagaharu. (1935). Genome analysis in *Brassica* with special reference to the experimental formation of *B. napus* and peculiar mode of fertilization. Jpn. J. Bot. 7, 389–452.

[B292] Van Berkum N. L. Lieberman-Aiden E. Williams L. Imakaev M. Gnirke A. Mirny L. A.. (2010). Hi-C: a method to study the three-dimensional architecture of genomes. J. Vis. Exp. 39:e1869. 10.3791/1869 20461051 PMC3149993

[B293] Van Dijk E. L. Jaszczyszyn Y. Naquin D. Thermes C. (2018). The third revolution in sequencing technology. Trends Genet. 34, 666–681. 10.1016/j.tig.2018.05.008 29941292

[B294] Varshney R. K. Pandey M. K. Bohra A. Singh V. K. Thudi M. Saxena R. K. (2018). Toward the sequence-based breeding in legumes in the post-genome sequencing era. Theor. Appl. Genet. 132, 797–816. 10.1007/s00122-018-3252-x 30560464 PMC6439141

[B295] Vernikos G. S. (2020). A review of pangenome tools and recent studies, in The Pangenome, eds Tettelin H. Medini D. (Cham: Springer), 89–112. 32633917

[B296] Voss-Fels K. Snowdon R. J. (2016). Understanding and utilizing crop genome diversity via high-resolution genotyping. Plant Biotechnol. J. 14, 1086–1094. 10.1111/pbi.12456 27003869 PMC11388962

[B297] Voytas D. F. Gao C. (2014). Precision genome engineering and agriculture: opportunities and regulatory challenges. PLoS Biol. 12:e1001877. 10.1371/journal.pbio.1001877 24915127 PMC4051594

[B298] Wan L. Li Y. Cen H. Zhu J. Yin W. Wu W.. (2018). Combining UAV-based vegetation indices and image classification to estimate flower number in oilseed rape. Remote Sens. 10:1484. 10.3390/rs10091484

[B299] Wang B. Kumar V. Olson A. Ware D. (2019). Reviving the transcriptome studies: an insight into the emergence of single-molecule transcriptome sequencing. Front. Genet. 1:384. 10.3389/fgene.2019.00384 31105749 PMC6498185

[B300] Wang G.-X. Lv J. Zhang J. Han S. Zong M. Guo N.. (2016). Genetic and epigenetic alterations of *Brassica nigra* introgression lines from somatic hybridization: a resource for cauliflower improvement. Front. Plant Sci. 7:1258. 10.3389/fpls.2016.01258 27625659 PMC5003894

[B301] Wang N. Shi L. Tian F. Ning H. Wu X. Long Y.. (2010). Assessment of *FAE1* polymorphisms in three *Brassica* species using EcoTILLING and their association with differences in seed erucic acid contents. BMC Plant Biol. 10:137. 10.1186/1471-2229-10-137 20594317 PMC3017795

[B302] Wang N. Wang Y. Tian F. King G. J. Zhang C. Long Y.. (2008). A functional genomics resource for *Brassica napus*: development of an EMS mutagenized population and discovery of *FAE1* point mutations by TILLING. New Phytol. 180, 751–765. 10.1111/j.1469-8137.2008.02619.x 18811617

[B303] Wang X. Wang H. Wang J. Sun R. Wu J. Liu S.. (2011). The genome of the mesopolyploid crop species *Brassica rapa*. Nat. Genet. 43, 1035–1039. 10.1038/ng.919 21873998

[B304] Wang X. Xu Y. Hu Z. Xu C. (2018). Genomic selection methods for crop improvement: current status and prospects. Crop J. 6, 330–340. 10.1016/j.cj.2018.03.001

[B305] Wang Y. Xiao L. Guo S. An F. Du D. (2016). Fine mapping and whole-genome resequencing identify the seed coat color gene in *Brassica rapa*. PLoS ONE 11:e0166464. 10.1371/journal.pone.0166464 27829069 PMC5102352

[B306] Wang Z. Wu X. Wu Z. An H. Yi B. Wen J.. (2018). Genome-wide DNA methylation comparison between *Brassica napus* genic male sterile line and restorer line. Int. J. Mol. Sci. 19:2689. 10.3390/ijms19092689 30201884 PMC6165103

[B307] Watanabe K. Sassa Y. Suda E. Chen C.-H. Inaba M. Kikuchi A. (2005). Global political, economic, social and technological issues on transgenic crops. Plant Biotechnol. 22, 515–522. 10.5511/plantbiotechnology.22.515

[B308] Weigel D. Colot V. (2012). Epialleles in plant evolution. Genome Biol. 13:249. 10.1186/gb-2012-13-10-249 23058244 PMC3491404

[B309] Werner C. R. Qian L. Voss-Fels K. P. Abbadi A. Leckband G. Frisch M.. (2017). Genome-ide regression models considering general and specifc combining ability predict hybrid performance in oilseed rape with similar accuracy regardless of trait architecture. Theor. Appl. Genet. 131, 299–317. 10.1007/s00122-017-3002-5 29080901

[B310] Werner C. R. Voss-Fels K. P. Miller C. N. Qian W. Hua W. Guan. (2018). Effective genomic selection in a narrow-genepool crop with low-density markers: Asian rapeseed as an example. Plant Genome 11:170084. 10.3835/plantgenome2017.09.0084 30025015 PMC12810071

[B311] White P. J. Broadley M. R. (2005). Historical variation in the mineral composition of edible horticultural products. J. Hortic. Sci. Biotechnol. 80, 660–667. 10.1080/14620316.2005.11511995

[B312] Wicke S. Schneeweiss G. M. dePamphilis C. W. Müller K. F. Quandt D. (2011). The evolution of the plastid chromosome in land plants: gene content, gene order, gene function. Plant Mol. Biol. 76, 273–297. 10.1007/s11103-011-9762-4 21424877 PMC3104136

[B313] Wolfram K. Schmidt J. Wray V. Milkowski C. Schliemann W. Strack D. (2010). Profiling of phenylpropanoids in transgenic low-sinapine oilseed rape (*Brassica napus*). Phytochemistry 71, 1076–1084. 10.1016/j.phytochem.2010.04.00 20451226

[B314] Wu D. Liang Z. Yan T. Xu Y. Xuan L. Tang J.. (2019). Whole-genome resequencing of a worldwide collection of rapeseed accessions reveals the genetic basis of ecotype divergence. Mol. Plant. 12, 30–43. 10.1016/j.molp.2018.11.007 30472326

[B315] Wu G. Truksa M. Datla N. Vrinten P. Bauer J. Zank T.. (2005). Stepwise engineering to produce high yields of very long-chain polyunsaturated fatty acids in plants. Nat. Biotechnol. 23, 1013–1017. 10.1038/nbt1107 15951804

[B316] Wu J. Chen C. Xian G. Liu D. Lin L. Yin S.. (2020a). Engineering herbicide-resistant oilseed rape by CRISPR/Cas9-mediated cytosine base-editing. Plant Biotechnol. J. 18, 1857–1859. 10.1111/pbi.13368 32096325 PMC7415781

[B317] Wu J. Liu B. Cheng F. Ramchiary N. Choi S.R. Lim Y.P.. (2012). Sequencing of chloroplast genome using whole cellular DNA and Solexa sequencing technology. Front. Plant Sci. 3:243. 10.3389/fpls.2012.00243 23162558 PMC3492724

[B318] Wu J. Yan G. Duan Z. Wang Z. Kang C. Guo L.. (2020b). Roles of the *Brassica napus* DELLA Protein BnaA6.RGA, in modulating drought tolerance by interacting with the ABA signalling component BnaA10.ABF2. Front. Plant Sci. 11:577. 10.3389/fpls.2020.00577 32477388 PMC7240051

[B319] Wu J. Zhao Q. Yang Q. Liu H. Li Q. Yi X.. (2016). Comparative transcriptomic analysis uncovers the complex genetic network for resistance to *Sclerotinia sclerotiorum* in *Brassica napus*. Sci. Rep. 6:19007. 10.1038/srep19007 26743436 PMC4705546

[B320] Würschum T. Abel S. Zhao Y. (2014). Potential of genomic selection in rapeseed (*Brassica napus* L.) breeding. Plant Breed. 133, 45–51. 10.1111/pbr.12137

[B321] Xiao-Ming Z. Junrui W. Li F. Sha L. Hongbo P. Lan Q.. (2017). Inferring the evolutionary mechanism of the chloroplast genome size by comparing whole-chloroplast genome sequences in seed plants. Sci. Rep. 7:1555. 10.1038/s41598-017-01518-5 28484234 PMC5431534

[B322] Xie T. Chen X. Guo T. Rong H. Chen Z. Sun Q.. (2020). Targeted knockout of *BnTT2* homologues for yellow-seeded *Brassica napus* with reduced flavonoids and improved fatty acid composition. J. Agric. Food Chem. 68, 5676–5690. 10.1021/acs.jafc.0c01126 32394708

[B323] Xin Q. Wang X. Gao Y. Xu D. Xie Z. Dong F.. (2020). Molecular mechanisms underpinning the multiallelic inheritance of MS5 in *Brassica napus*. Plant J. Cell Mol. Biol. 103, 1723–1734. 10.1111/tpj.14857 32445599

[B324] Xiong X. Zhou D. Xu L. Liu T. Yue X. Liu W.. (2019). BcPME37c is involved in pollen intine formation in *Brassica campestris*. Biochem. Biophys. Res. Commun. 517, 63–68. 10.1016/j.bbrc.2019.07.009 31320138

[B325] Xue J.-Y. Wang Y. Chen M. Dong S. Shao Z.-Q. Liu Y. (2020). Maternal inheritance of U's triangle and evolutionary process of *Brassica* mitochondrial genomes. Front. Plant Sci. 11:805. 10.3389/fpls.2020.00805 32595682 PMC7303332

[B326] Yamagishi H. Tanaka Y. Terachi T. (2014). Complete mitochondrial genome sequence of black mustard (*Brassica nigra*; BB) and comparison with *Brassica oleracea* (CC) and *Brassica carinata* (BBCC). Genome 57, 577–582. 10.1139/gen-2014-0165 25767903

[B327] Yan C. Huang Y. Liu Z. Guo F. Jiao Z. Yang W.. (2020). Rapid identification of yellow-flowered gene *Bofc* in cauliflower (*Brassica oleracea* var. botrytis) by bulked segregant analysis and whole-genome resequencing. Euphytica 216:26. 10.1007/s10681-020-2560-9

[B328] Yan G. Nelson M. Pradhan A. Mason A. Weerakoon S. Si P.. (2009). Progress towards the creation of trigenomic brassica hexaploid populations. SABRAO, J. Breed. Genet. 41:00274.

[B329] Yang H. Wu J. Tang T. Liu K.-D. Dai C. (2017). CRISPR/Cas9-mediated genome editing efficiently creates specific mutations at multiple loci using one sgRNA in *Brassica napus*. Sci. Rep. 7:7489. 10.1038/s41598-017-07871-9 28790350 PMC5548805

[B330] Yang J. Liu D. Wang X. Ji C. Cheng F. Liu B.. (2016a). The genome sequence of allopolyploid *Brassica juncea* and analysis of differential homoeolog gene expression influencing selection. Nat. Genet. 48, 1225–1232. 10.1038/ng.3657 27595476

[B331] Yang J. Liu G. Zhao N. Chen S. Liu D. Ma W.. (2016b). Comparative mitochondrial genome analysis reveals the evolutionary rearrangement mechanism in *Brassica*. Plant Biol. 18, 527–536. 10.1111/plb.12414 27079962

[B332] Yang K. Nath U. K. Biswas M. K. Kayum M. A. Yi G-e. Lee J.. (2018). Whole-genome sequencing of *Brassica oleracea* var. *capitata* reveals new diversity of the mitogenome. PLoS ONE 13:e0194356. 10.1371/journal.pone.0194356 29547671 PMC5856397

[B333] Yang R. Jarvis D. E. Chen H. Beilstein M. A. Grimwood J. Jenkins J.. (2013). The reference genome of the halophytic plant *Eutrema salsugineum*. Front. Plant Sci. 4:46. 10.3389/fpls.2013.00046 23518688 PMC3604812

[B334] Yang Y. Zhu K. Li H. Han S. Meng Q. Khan S. U.. (2018). Precise editing of CLAVATA genes in *Brassica napus* L. regulates multilocular silique development. Plant Biotechnol. J. 16, 1322–1335. 10.1111/pbi.12872 29250878 PMC5999189

[B335] Yao M. Guan M. Zhang Z. Cui Y. Chen H. Liu W.. (2020). GWAS and co-expression network combination uncovers multigenes with close linkage effects on the oleic acid content accumulation in *Brassica napus*. BMC Genomics 21:320. 10.1186/s12864-020-6711-0 32326904 PMC7181522

[B336] You Q. Yang X. Peng Z. Xu L. Wang J. (2018). Development and applications of a high throughput genotyping tool for polyploid crops: single nucleotide polymorphism (SNP) array. Front. Plant Sci. 9:104. 10.3389/fpls.2018.00104 29467780 PMC5808122

[B337] Yousef E. Müller T. Börner A. Schmid K. J. (2018). Comparative analysis of genetic diversity and differentiation of cauliflower (*Brassica oleracea* var. *botrytis)* accessions from two *ex situ* genebanks. PLoS ONE 13:e0192062. 10.1371/journal.pone.0192062 29420661 PMC5805252

[B338] Yu J. Yang X. D. Wang Q. Gao L. W. Yang Y. Xiao D.. (2018). Efficient virus-induced gene silencing in *Brassica rapa* using a turnip yellow mosaic virus vector. Biol. Plant. 62, 826–834. 10.1007/s10535-018-0803-6

[B339] Zaman Q. U. Chu W. Hao M. Shi Y. Sun M. Sang S. F.. (2019). CRISPR/Cas9-mediated multiplex genome editing of JAGGED gene in *Brassica napus* L. Biomolecules 9:725. 10.3390/biom9110725 31726660 PMC6921047

[B340] Zarinpanjeh N. Motallebi M. Zamani M. R. Ziaei M. (2016). Enhanced resistance to *Sclerotinia sclerotiorum* in *Brassica napus* by co-expression of defensin and chimeric chitinase genes. J. Appl. Genet. 57:417–425. 10.1007/s13353-016-0340-y 26862081

[B341] Zeng C.-L. Wang G.-Y. Wang J.-B. Yan G.-X. Chen B.-Y. Xu K.. (2012). High-throughput discovery of chloroplast and mitochondrial DNA polymorphisms in *Brassica*ceae species by ORG-EcoTILLING. PLoS ONE 7:e47284. 10.1371/journal.pone.0047284 23185237 PMC3504036

[B342] Zhai Y. Cai S. Hu L. Yang Y. Amoo O. Fan C.. (2019). CRISPR/Cas9-mediated genome editing reveals differences in the contribution of *INDEHISCENT* homologues to pod shatter resistance in *Brassica napus* L. Theor. Appl. Genet. 132, 2111–2123. 10.1007/s00122-019-03341-0 30980103

[B343] Zhai Y. Yu K. Cai S. Hu L. Amoo O. Xu L.. (2020). Targeted mutagenesis of *BnTT8* homologs controls yellow seed coat development for effective oil production in *Brassica napus* L. Plant Biotech J. 18, 1153–1168. 10.1111/pbi.13281 31637846 PMC7152602

[B344] Zhang A. Wang H. Beyene Y. Samagn K. Liu Y. Cao S.. (2017). Effect of trait heritability, training population size and marker density on genomic prediction accuracy estimation in 22 bi-parental tropical maize populations. Front. Plant Sci. 8:1916. 10.3389/fpls.2017.01916 29167677 PMC5683035

[B345] Zhang C. Wohlhueter R. Zhang H. (2016). Genetically modified foods: a critical review of their promise and problems. Food Sci. Hum. Wellness 5, 116–123. 10.1016/j.fshw.2016.04.002

[B346] Zhang F. Batley J. (2020). Exploring the application of wild species for crop improvement in a changing climate. Curr. Opin. Plant Biol. 56, 218–222. 10.1016/j.pbi.2019.12.013 32029361

[B347] Zhang K. Nie L. Cheng Q. Yin Y. Chen K. Qi F.. (2019). Effective editing for lysophosphatidic acid acyltransferase 2/5 in allotetraploid rapeseed (*Brassica napus* L.) using CRISPR-Cas9 system. Biotechnol. Biofuels 12:225. 10.1186/s13068-019-1567-8 31548867 PMC6753616

[B348] Zhang L. Cai X. Wu J. Liu M. Grob S. Cheng F.. (2018). Improved *Brassica rapa* reference genome by single-molecule sequencing and chromosome conformation capture technologies. Hortic. Res. 5:50. 10.1038/s41438-018-0071-9 30131865 PMC6092429

[B349] Zhang T. Hu Y. Jiang W. Fang L. Guan X. Chen J.. (2015). Sequencing of allotetraploid cotton (*Gossypium hirsutum* L. acc. TM-1) provides a resource for fiber improvement. Nat. Biotechnol. 33, 531–537. 10.1038/nbt.3207 25893781

[B350] Zhang Y. Huang S. Wang X. Liu J. Guo X. Mu J.. (2018a). Defective APETALA2 genes lead to sepal modification in *Brassica crops*. Front. Plant Sci. 9:367. 10.3389/fpls.2018.00367 29616073 PMC5869249

[B351] Zhang Y. Massel K. Godwin I. D. Gao G. (2018b). Applications and potential of genome editing in crop improvement. Genome Biol. 19:210. 10.1186/s13059-018-1586-y 30501614 PMC6267055

[B352] Zhang Y. Thomas W. Bayer P. E. Edwards D. Batley J. (2020). Frontiers in dissecting and managing *Brassica* diseases: from reference-based RGA candidate identification to building pan-RGAomes. Int. J. Mol. Sci. 21:8964. 10.3390/ijms21238964 33255840 PMC7728316

[B353] Zhang Z. Zhang S. Huang X. Orwig K. E. Sheng Y. (2013). Rapid assembly of customized TALENs into multiple delivery systems. PLoS ONE 8:e80281. 10.1371/journal.pone.0080281 24244669 PMC3820630

[B354] Zhao Y. Mette M. F. Gowda M. Longin C. F. H. Reif J. C. (2014). Bridging the gap between marker-assisted and genomic selection of heading time and plant height in hybrid wheat. Heredity 112, 638–645. 10.1038/hdy.2014.1 24518889 PMC4023446

[B355] Zhao Y. S. Mette M. F. Reif J. C. (2015). Genomic selection in hybrid breeding. Plant Breed. 134, 1–10. 10.1111/pbr.12231

[B356] Zheng M. Zhang L. Tang M. Liu J. Liu H. Yang H.. (2020). Knockout of two *BnaMAX1* homologs by CRISPR/Cas9-targeted mutagenesis improves plant architecture and increases yield in rapeseed (*Brassica napus* L.). Plant Biotech. J. 18, 644–654. 10.1111/pbi.13228 31373135 PMC7004912

[B357] Zhou J. Chen T. Cheng C. Xianhong G. Zaiyun L. (2016). Distinct subgenome stabilities in synthesized *Brassica* allohexaploids. Theor. Appl. Genet. 129, 1257–1271. 10.1007/s00122-016-2701-7 26971112

[B358] Zou J. Mao L. Qiu J. Wang M. Jia L. Wu D.. (2019). Genome-wide selection footprints and deleterious variations in young Asian allotetraploid rapeseed. Plant Biotechnol. J. 17, 1998–2010. 10.1111/pbi.13115 30947395 PMC6737024

[B359] Zou J. Y Zhao P. Liu L. Shi X. Wang M.. (2016). Seed quality traits can be predicted with high accuracy in *Brassica napus* using genomic data. PLoS ONE 11:e0166624. 10.1371/journal.pone.0166624 27880793 PMC5120799

